# Immune‐related signature predicts the prognosis and immunotherapy benefit in bladder cancer

**DOI:** 10.1002/cam4.3400

**Published:** 2020-08-25

**Authors:** Yejinpeng Wang, Liang Chen, Mengxue Yu, Yayun Fang, Kaiyu Qian, Gang Wang, Lingao Ju, Yu Xiao, Xinghuan Wang

**Affiliations:** ^1^ Department of Urology Zhongnan Hospital of Wuhan University Wuhan China; ^2^ Department of Biological Repositories Zhongnan Hospital of Wuhan University Wuhan China; ^3^ Human Genetics Resource Preservation Center of Hubei Province Wuhan China; ^4^ Human Genetics Resource Preservation Center of Wuhan University Wuhan China; ^5^ Laboratory of Precision Medicine Zhongnan Hospital of Wuhan University Wuhan China; ^6^ Medical Research Institute Wuhan University Wuhan China

**Keywords:** bladder cancer (BCa), macrophage, programmed death ligand‐1 (PD‐L1), regulatory T cells (Tregs), transforming growth factor β (TGF‐β), tumor microenvironment (TME)

## Abstract

**Background:**

There is no good prognostic model that could predict the prognosis of bladder cancer (BCa) and the benefit of immunotherapy.

**Methods:**

Through the least absolute shrinkage and selection operator (LASSO) algorithm, we constructed a 13‐mRNA immune signature from the TCGA cohort (n = 406). We validated its prognostic value and predictive value for the benefit of immunotherapy with four independent validation cohort (GSE13507 [n = 256], GSE31684 [n = 93], GSE32894 [n = 308], and IMvigor210 cohort [n = 298]).

**Results:**

Our results indicating that high‐risk group with higher inhibitory immune cell infiltration (regulatory T cells [Tregs] and macrophage, etc), higher expression of immune checkpoints, and more T cell suppressive pathways (transforming growth factor β [TGF‐β], epithelial‐mesenchymal transition [EMT], etc) were activated. Besides, the immune signature showed a good predictive value for the benefit of immunotherapy in a cohort of urothelial carcinoma patients treated with PD‐L1.

**Conclusions:**

The immune signature constructed is convenient to classify the immunotherapeutic susceptibility of patients with BCa, so as to achieve precision immunotherapy for BCa.

## Graphical abstract

High‐risk group with higher inhibitory immune cell infiltration, higher expression of immune checkpoints, and more T cell suppressive pathways was activated, and these together contribute to immune escape and might be the cause of poor prognosis. Besides, the immune signature showed a good predictive value for the benefit of immunotherapy in a cohort of urothelial carcinoma patients treated with PD‐L1.

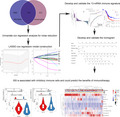

**Abbreviations**
BCa: bladder cancer
CTLA4: cytotoxic T‐lymphocyte‐associated protein 4
EMT: epithelial‐mesenchymal transition
FPKM: Fragments Per Kilobase Million
LASSO: Least Absolute Shrinkage and Selection Operator
MIBC: muscle‐invasive bladder cancer
NMIBC: nonmuscle‐invasive bladder cancer
PD‐L1: programmed death ligand‐1
ssGSEA: single sample Gene Set Enrichment Analysis
TGF‐β: transforming growth factor β
TME: tumor microenvironment
TPM: Transcripts Per Million
Tregs: regulatory T cells

## 1 INTRODUCTION

Bladder cancer (BCa) is the ninth most common cancer worldwide and the 13th most fatal. Smoking remains the most associated risk factor for BCa.^1^ Bacillus Calmette‐Guérin (BCG) is one of the most successful immunotherapies for BCa. According to the existing research, the antitumor effect of BCG is mainly attributed to the synergistic effect of urothelial cells and immune system cells.^2^ In recent years, the treatment landscape of BCa has been changed by the introduction of checkpoint blockade (CPB).^3^, ^4^ However, many patients currently treated with immune CPB do not benefit very well, and there are no unique biomarkers that could predict patients’ treatment benefits.^5^ According to the anatomy, BCa can be divided into nonmuscle‐invasive bladder cancer (NMIBC) and muscle‐invasive bladder cancer (MIBC).^6^, ^7^ NMIBC has a high recurrence rate and progression rate,^8^ while MIBC has a high mortality rate.^9^, ^10^ Similar to immune benefit, there are no good biomarkers or prognostic signature to accurately predict the prognosis of BCa.

As tumors develop, the body's immune system is activated to resist tumor development. Unfortunately, tumor cells employ various strategies to delay or even stop the body's immune system from suppressing tumors, a phenomenon known as immune escape.^11^ The occurrence of immune escape usually leads to malignant progression, metastasis, poor prognosis, and failure of immunotherapy. Several immune escape mechanisms have been identified, one of the key mechanisms is that tumor cells induce and recruit inhibitory immune cells (Tregs, macrophages, etc) to promote immune escape.^12^ Immune checkpoints, such as programmed cell death protein‐1 (PD‐1), programmed death ligand‐1 (PD‐L1), and cytotoxic T‐lymphocyte‐associated protein 4 (CTLA4), are responsible for immune escape.^13^, ^14^ Also contributing to immune escape are cytokines such as vascular endothelial growth factor (VEGF) and transforming growth factor β (TGF‐β).^15^, ^16^

With the maturity of high‐throughput technology and the development of many algorithms aimed at studying tumor immunity (single sample Gene Set Enrichment Analysis [ssGSEA], quanTIseq, CIBERSORT, etc),^17^, ^18^, ^19^ it is possible to describe the landscape of tumor immune microenvironment (TME) through transcriptome sequencing.^20^, ^21^ However, no studies have systematically delineated the TME in BCa. We constructed an immune signature of 13‐mRNA based on the least absolute shrinkage and selection operator (LASSO) algorithm and then, the TME landscape of BCa was depicted according to two currently popular algorithms and quantitatively estimated the main immune cells, thus finding a significant correlation between immune signature score (ISS) and immune escape phenotype. Based on this immune signature, we could easily predict the prognosis and benefit of immunotherapy in BCa patients.

## 2 MATERIALS AND METHODS

### 2.1 Data collection and preprocessing

The Cancer Genome Atlas (TCGA) level 4 RNA‐sequencing (RNA‐seq) data (Fragments Per Kilobase Million [FPKM] values and count values) were downloaded from the UCSC Xena website (https://gdc.xenahubs.net). In order to make the RNA‐seq data more comparable with the microarray data,^22^ we converted the FPKM values to the Transcripts Per Million (TPM) values. For somatic mutation data, we downloaded the MuTect2 dataset (SNPs and small INDELs, and MuTect2 Variant Aggregation and Masking) from the same website, and synonymous mutations and mutations with a mutation frequency of less than 5% were removed. Prognostic data for all TCGA survival analyses were obtained from published paper.^23^

For the Affymetrix microarray data of the Gene Expression Omnibus database (GEO, https://www.ncbi.nlm.nih.gov/geo/), the RMA algorithm of R package “affy”^24^ was applied for background adjustment and normalization, and then logarithmic processing. For the Illumina data, we followed the “lumi” software's protocol to perform preprocessing.

The IMvigor210 cohort was downloaded from the website http://research‐pub.gene.com/IMvigor210CoreBiologies, which was a cohort study of atezolizumab in patients with locally advanced or metastatic urothelial carcinoma.^15^ For the microarray data of this cohort, the R package “arrayQualityMetrics” was used to quality control, and the trimmed mean of M‐values was used to normalize the count data. The following logarithmic processing was carried out through “voom” function of R package “limma”.^25^, ^26^ Samples from IMvigor210 cohort that had no clinical response were removed.

**FIGURE 1:**
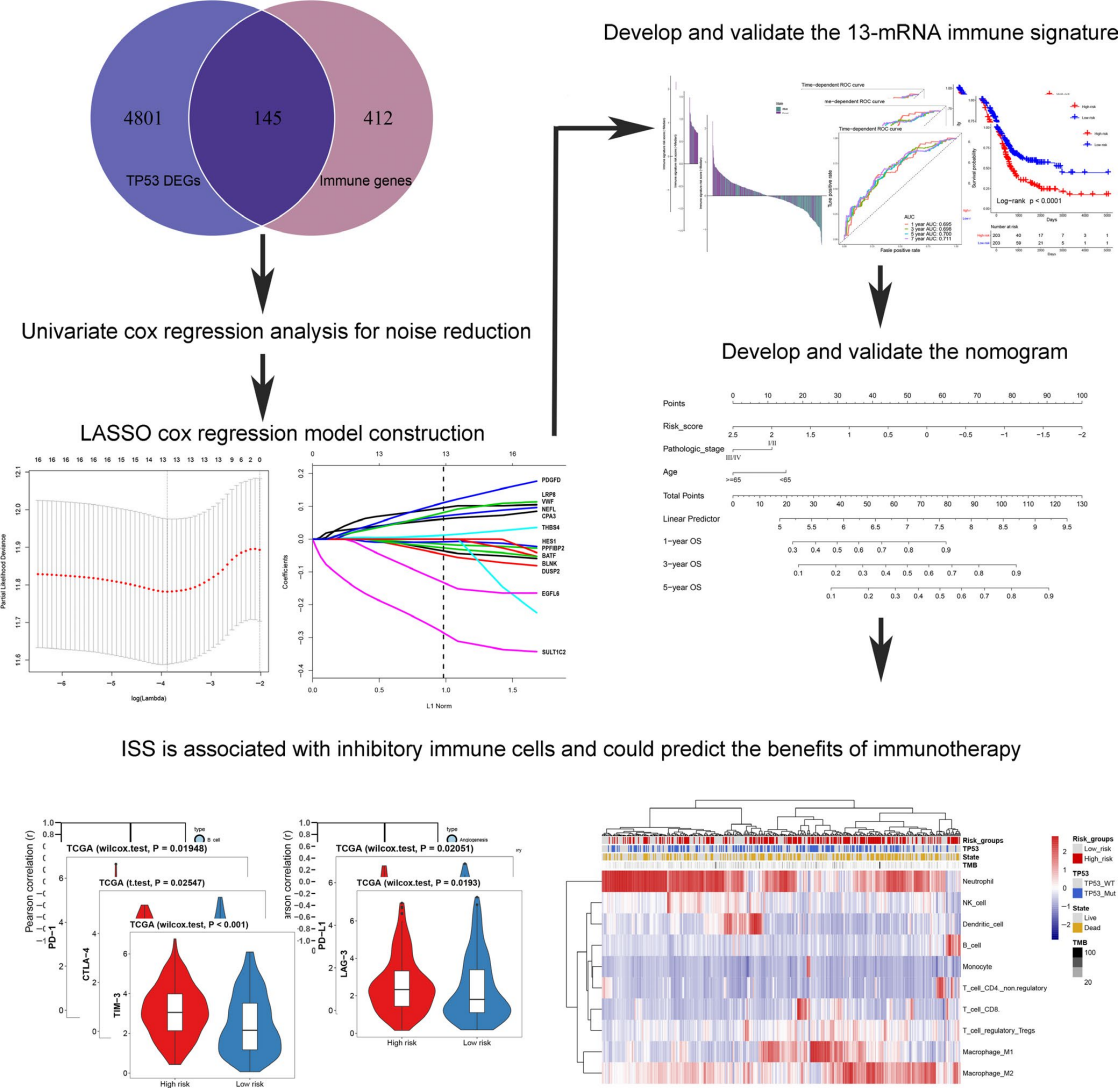
Flow chart of the study. LASSO, Least absolute shrinkage and selection operator; ISS, immune signature score

For all cohorts, only samples containing prognostic data were retained. The details of all the cohorts used are listed in Table 1, and the flow chart of our entire study is shown in Figure 1.

**TABLE 1:** Details of the cohort used

Series accession numbers	GSE13507	GSE31684	GSE32894	TCGA‐BLCA	IMvigor210
Platform	Illumina human‐6 v2.0 expression beadchip	Affymetrix Human Genome U133 Plus 2.0 Array	Illumina HumanHT‐12 V3.0 expression beadchip	Illumina RNA‐seq	Illumina HiSeq 2500
No. of patients	256	93	308	406	298
Subtype	NMIBC: 103; MIBC: 62	NMIBC: 15; MIBC: 78	NMIBC: 215; MIBC: 93	NMIBC: 5; MIBC: 401	
Gender	Female: 30, Male: 135	Female: 25, Male: 68	Female: 80, Male: 228	Female: 107, Male: 299	Female: 65, Male: 233
Survival outcome	OS, PFS	OS, RFS	DFS	OS, PFS	OS

### 2.2 Differentially expressed gene screening

To construct an immune‐related prognostic signature, we referred to the method that has been published,^27^ and calculated the differentially expressed genes (DEGs) between TP53 mutation type (TP53^Mut^) and TP53 wild‐type (TP53^WT^) samples. We used the count value as the input matrix, and the R package “DESeq2”^28^ was applied to perform DEGs analysis. We used the false discovery rate (FDR) <0.05 as the screening threshold.

### 2.3 Dimension reduction and establishment of the immune signature

We intersected the DEGs and immune‐related genes^29^ obtained in the previous step, then univariate analysis was used to perform the dimension reduction to reduce the noise of gene without prognostic value (*P* < .05 was considered to have prognostic value). We randomly divided the data from TCGA cohort into two groups according to the ratio of 3:1, the former as the training cohort (n = 304), the latter as the internal validation cohort (n = 102), and all the samples as the entire cohort (n = 406). The R packet “LASSO”^30^ was used to establish the immune signature on the training cohort. The LASSO algorithm could reduce the dimension of high‐latitude data,^31^ and the degree of high‐latitude data complexity was controlled by the parameter *λ*, the larger *λ* was, the greater the penalty was, so as to get a model with fewer variables.^32^ We ran 10‐fold cross‐validation through the function “cv.glmnet” to get a stable model. Finally, Cox regression coefficient calculated by LASSO algorithm was used to construct the immune signature, and ISS was defined as $\text{ISS}=\sum \beta_{i}G_{i}$, where *β* is the Cox regression coefficient of the mRNA; and *G* is the mRNA expression value of gene *i*. According to the median of ISS, we divided the patients into high‐risk and low‐risk groups. The time‐dependent ROC curve performed by R package “survivalROC”^33^ and Kaplan‐Meier survival curve analysis performed by R package “survival” both were used to verify the accuracy of the prognostic value of the immune signature. To avoid the overfitting effect, we validated the prognostic value of immune signature with two internal validation cohorts (TCGA internal validation cohort and entire validation cohort) and three independent external validation cohorts (GSE13057, GSE31684, and GSE32894).

### 2.4 Quantitative estimation of immune cell infiltration

The “quanTIseq” algorithm was used to quantify the infiltration of immune cells in BCa. “QuanTIseq” is an algorithm for quantifying tumor immune infiltration from human RNA‐seq data, and it quantifies the proportions of 10 different immune cell types (neutrophil, NK cell, B cell, T cell CD4, macrophage, etc) by deconvolution. This algorithm could estimate the absolute proportion of related immune cell types from RNA‐seq data, thus supports comparisons between and within samples, which some current deconvolution algorithms cannot do (such as CIBERSORT, TIMER, EPIC).^18^ Following the protocol of the R package “immunedeconv”,^34^ we used a matrix of TPM values (no logarithmic processing) as input to perform the deconvolution. The R package “immunedeconv” was downloaded from https://github.com/grst/immunedeconv. In addition, we used the R package “estimate”^35^ to infer the fraction of immune and stromal cells in tumor samples, this package was based on ssGSEA algorithm, from a macro perspective to detect the distribution of immune and stromal infiltration.

### 2.5 Functional and pathway annotation of the immune signature

To find the functions and pathways represented by the immune signature, we calculated the correlation coefficients between all genes and ISS. Gene Ontology (GO) and Kyoto Encyclopedia of Genes and Genomes (KEGG) analysis were performed through the R package “ClusterProfiler”.^36^ All genes were sequenced according to the magnitude of the correlation coefficient, so as to conduct gene set enrichment analysis (GSEA), and *P* < .05 was considered significant.

To understand the correlation between immune signature and other immune‐related pathways, we first convert the logarithmic TPM value to a z‐score using the R package “GSVA”,^37^ and published immune pathway signature to conduct ssGSEA analysis of the immune signature.

### 2.6 Statistical analysis

For the comparison of two groups of data, if the data are normally distributed, unpaired *t*‐student test was used to compare the difference between them. If the data were non‐normally distributed variables, a nonparametric test (Wilcoxon rank‐sum test) was selected. For more than two groups, Kruskal‐Wallis test or one‐way analysis of variance was selected. All the calculations of correlation were computed by the “Pearson” algorithm. The univariate and multivariate Cox regression analyses were performed by R package “survival”, and the R package “forestplot” was used to visualize the result. Based on the results of multivariate analysis, we used prognostic factors that were independent of other factors to construct a nomogram through the R package “rms”. The calibration curve was used to test the accuracy of the nomogram. If the degree of fitting between the observed value and the actual value is higher, it indicates that the nomogram's prediction accuracy is higher. The decision curve analysis (DCA) performed by R package “rmda” was used to determine if the predictive model was clinically useful.^38^ The logistic regression and receiver operating characteristic (ROC) were used to measure the predictive value of tumor mutational burden (TMB) and/or ISS for immunosuppressive benefits. *P* < .05 was considered statistically significant. All statistical calculations were performed on R software (3.6.1 version).

## 3 RESULTS

### 3.1 Screening differentially expressed immune‐related gene

The R package “DESeq2” was used to screen DEGs between TP53^Mut^ and TP53^WT^ samples in TCGA‐BLCA cohort; a total of 4946 DEGs were identified, under the threshold of FDR <0.05. Intersections with published immune‐related genes,^29^ 145 candidate genes, were obtained (Figure 1).

### 3.2 Construction of immune signature

**FIGURE 2:**
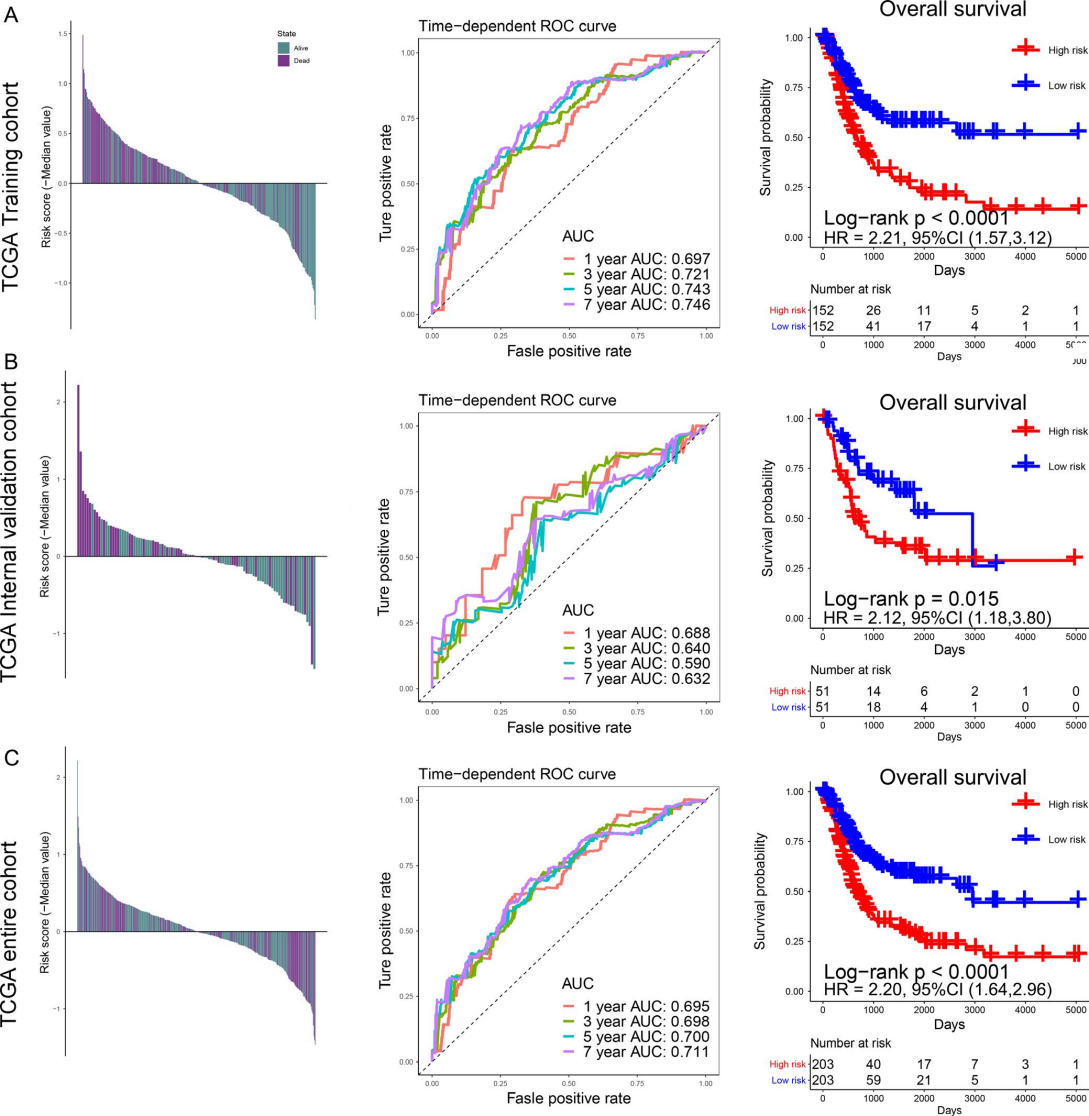
Risk scores by the immune signature, the time‐dependent ROC curves, and Kaplan‐Meier survival curves in the TCGA training cohort, internal validation cohort, and entire cohort. (A) Training cohort. (B) Internal validation cohort. (C) Entire cohort. The panels on the left represent the distribution of the survival status of each patient based on ISS; the middle panels indicate the time‐dependent ROC curve of each cohort, the AUCs at 1, 3, 5, and 7 years were used to assess the prognostic accuracy; the right panels represent the Kaplan‐Meier survival curve, the log‐rank test was used to calculated the *P* values. AUC, Area Under Curve; HR, Hazard Ratio; CI, Confidence Interval

**FIGURE 3:**
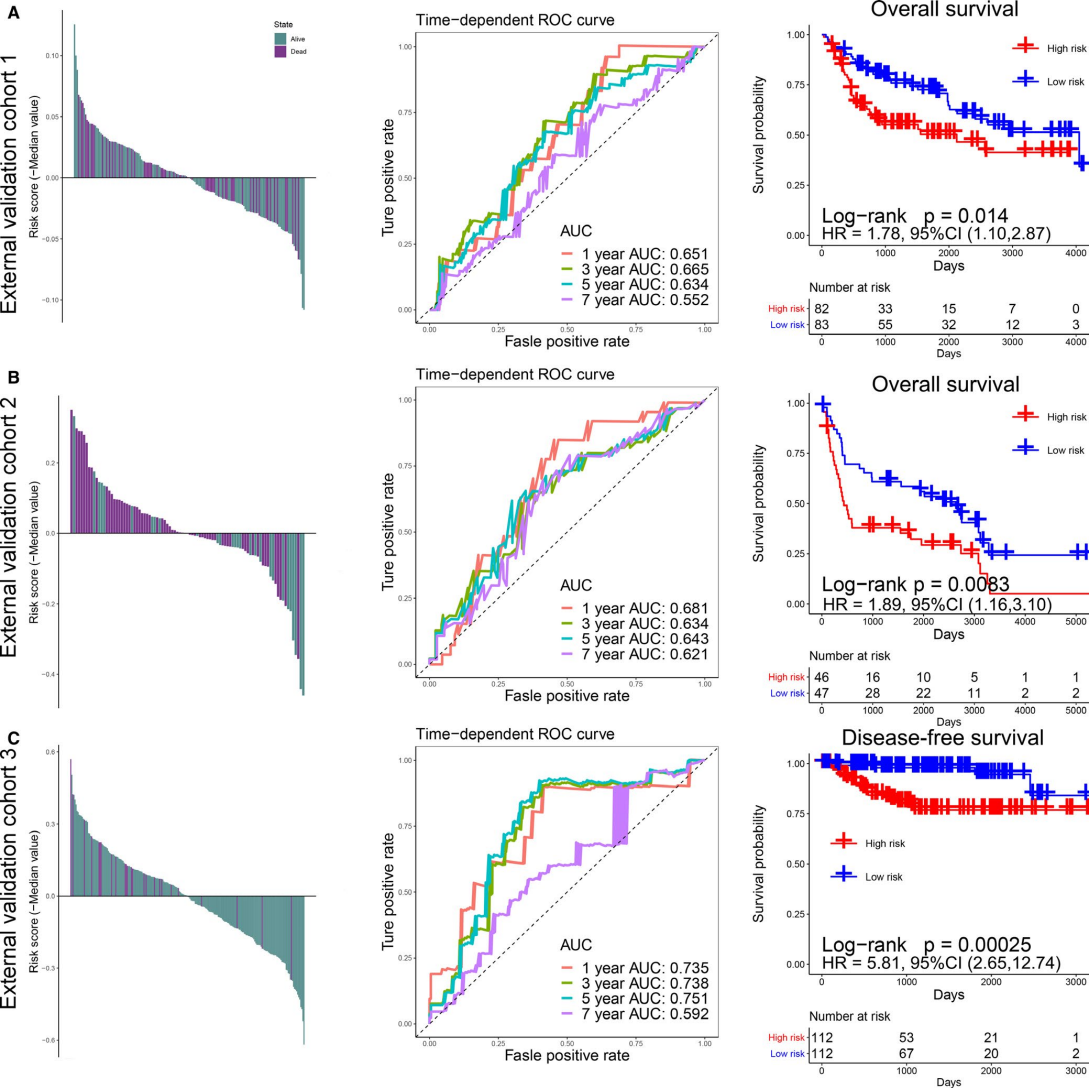
Risk scores by the immune signature, the time‐dependent ROC curves, and Kaplan‐Meier survival curves in the external validation cohorts 1, 2, and 3. (A) GSE13507 cohort. (B) GSE31684 cohort. (C) GSE32894 cohort. AUC, Area Under Curve; HR, Hazard Ratio; CI, Confidence Interval

We obtained immune‐related genes with prognostic value after noise reduction treatment using univariate regression analysis. The R package of “glmnet” was used to construct LASSO Cox regression analysis. After 1000 times 10 cross‐validation, we obtained an optimal *λ* value of 0.023 and finally, we established an immune signature based on 13‐mRNA (Figure 1, Table S1). We divided the patients into high‐ and low‐risk groups based on the median ISS (Figures 2, 3, left panels). We used TCGA training cohort (HR = 2.21, 95% CI: 1.57‐3.12, *P* < .001, Figure 2A) and two internal validation cohorts (TCGA internal validation cohort: HR = 2.12, 95% CI: 1.18‐3.80, *P* = .015; TCGA entire cohort: HR = 2.20, 95% CI: 1.64‐2.96, *P* < .001, Figure 2B,C) and three independent cohorts (GSE13507: HR = 1.78, 95% CI: 1.10‐2.87, *P* = .014; GSE31684: HR = 1.89, 95% CI: 1.16‐3.10, *P* = .083; GSE32894: HR = 5.81, 95% CI: 2.65‐12.74, *P* < .001, Figure 3A‐C) to verify the prognostic value of this immune signature. The result of time‐dependent ROC curve analysis indicated that the immune signature could accurately predict the overall survival (OS) (Figures 2 and 3A‐B) and PFS (Figure 3C, Figure S1A‐C) of BCa patients. Kaplan‐Meier survival curve analysis indicated that the high‐risk subtype had a significantly worse prognosis (Figures 2, 3, right panels). We explored the prognostic value of the immune signature in different stages and grades, and the results indicated that the prognostic signature basically performed well in each subgroup (Figure S1D‐F). Since this immune signature was constructed based on DEGs of the TP53 mutation state, we explored the prognostic value of the immune signature of different TP53 mutation state isoforms, and found that immune signature exhibited good prognostic value in both TP53 mutant and wild‐type patients (Figure S1G‐H).

### 3.3 Cox regression analysis and the construction of nomogram

**FIGURE 4:**
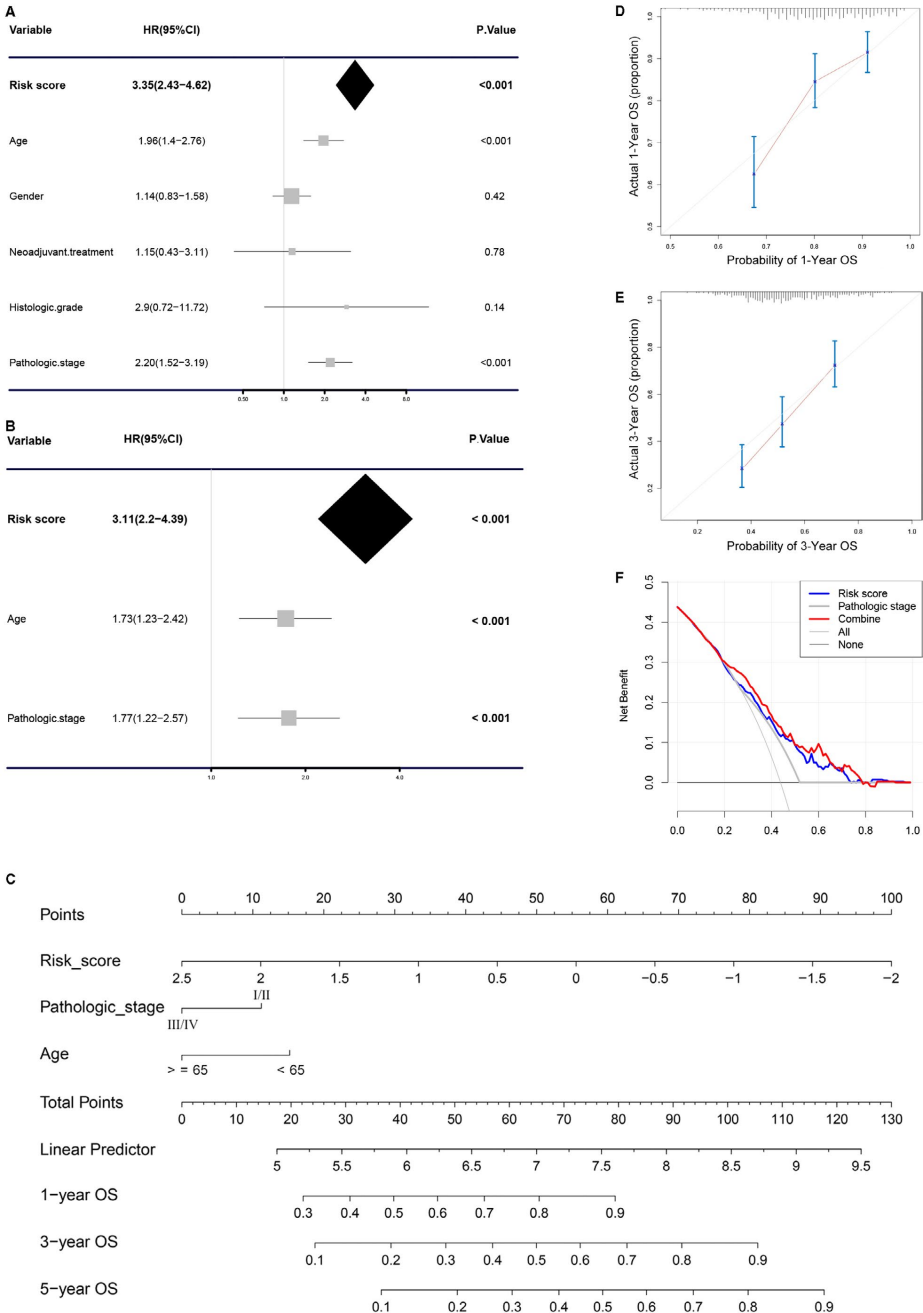
Cox regression analysis and establishment of nomogram. Univariable analysis (A) and multivariable analysis (B) of the risk score, age, gender, histologic grade, and so on. (C) The nomogram for predicting the proportion of BCa patients with 1‐, 3‐, or 5‐year overall survival (OS). Plots depict the calibration of the nomogram between predicted and observed (D) 1‐ or (E) 3‐year outcomes. (F) Decision curve analysis was used to evaluate the clinical utility of nomogram

To explore whether this immune signature could predict prognosis independently of other factors, Cox regression analysis was performed. We first performed univariate analysis of immune signature and other potentially prognostic factors (age [≥65 vs <65], gender [female vs male], neoadjuvant treatment [yes vs no], histologic grade [high vs low], pathologic stage [Ⅲ/Ⅳ vs Ⅰ/Ⅱ]), and the results indicated that the immune signature (HR = 3.35, 95% CI: 2.43‐4.62, *P* < .001), age (HR = 1.96, 95% CI: 1.40‐2.76, *P* < .001), and pathologic stage (HR = 2.20, 95% CI: 1.52‐3.19, *P* < .001) had high prognostic value (Figure 4A). Then, we performed multivariate Cox regression analysis of them, and the results showed that the immune signature (HR = 3.11, 95% CI: 2.20‐4.39, *P* < .001), age (HR = 1.73, 95% CI: 1.23‐2.42, *P* < .001), and pathologic stage (HR = 1.77, 95% CI: 1.22‐2.57, *P* < .001) could predict the prognosis of BCa independently of other factors (Figure 4B).

Since such results were not convenient for clinicians to predict the prognosis of patients with BCa, we constructed a nomogram based on the results of multivariate Cox regression analysis (Figure 4C). The ISS, age, and stage were scored separately, and finally, the comprehensive score was combined with the three, which was convenient to predict the 1‐, 3‐, and 5‐year survival rate of BCa. In order to verify the reliability of the nomogram, we verified the nomogram with the calibration curve and DCA. The results of calibration curve indicated that this immune signature was highly consistent with the ideal model (the blue line in the figure is basically the same as the 45° gray line. Figure 4D,E, Figure S1I); the results of DCA curve indicated that the nomogram showed high clinical application potential and net benefits (Figure 4F).

### 3.4 The landscape of the TME in bladder cancer

**FIGURE 5:**
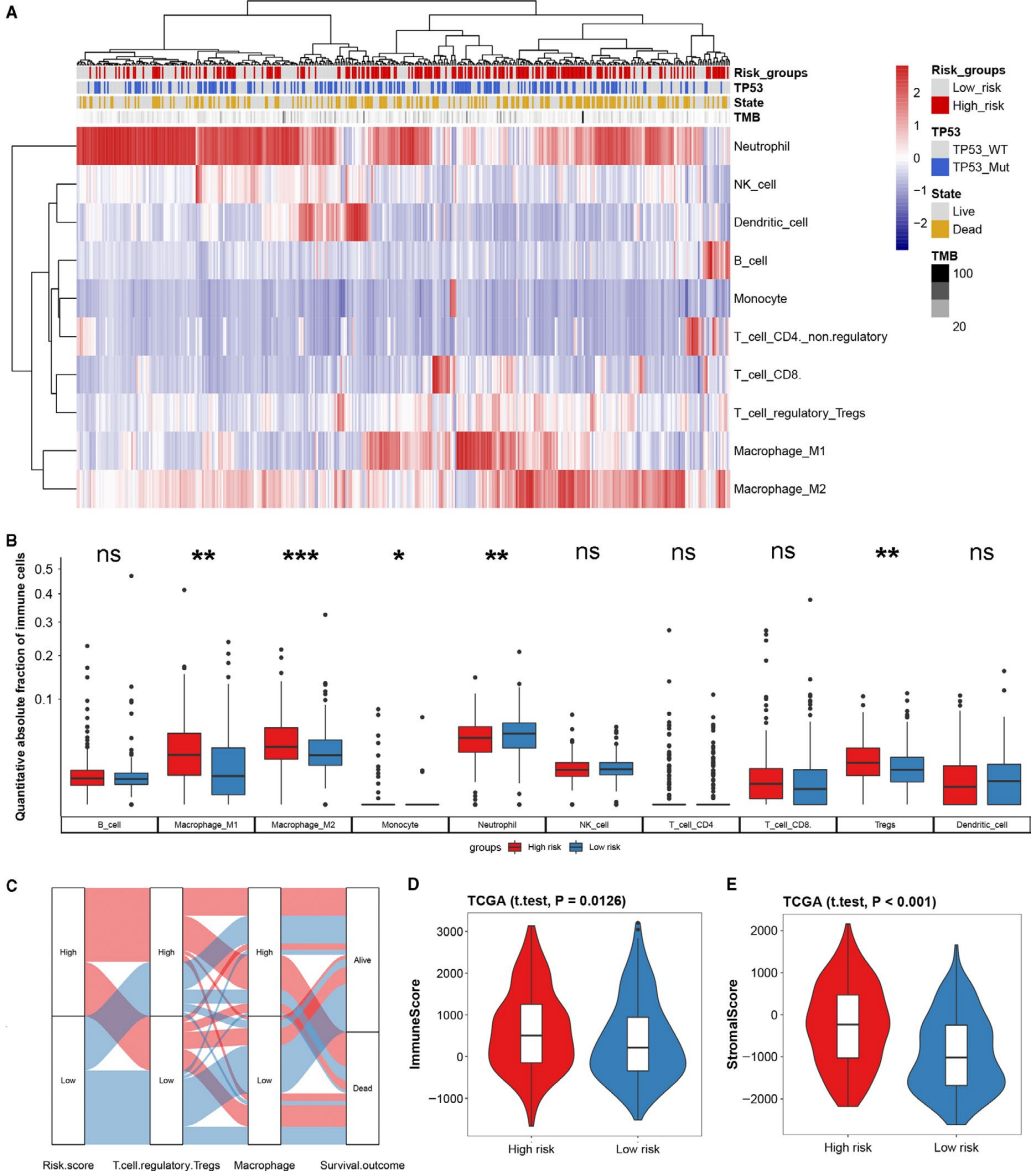
Landscape of tumor immune microenvironment based on the immune signature. (A) Heatmap of absolute abundance of tumor immune cell infiltration, the risk group, TP53 mutation state, survival state, and tumor mutational burden (TMB) are shown as patient annotations. (B) Box plot of the distribution of 10 immune cells in the high‐ and low‐risk group; the statistical difference of two groups was compared through unpaired *t*‐test or Wilcoxon test. (C) Alluvial diagram of the immune signature in groups with different levels of ISS, Tregs, macrophage, and survival outcomes. Violin plots based on “estimate” algorithm for quantitative estimation of immune (D) and stromal (E) infiltration between the high‐ and low‐risk groups. *P < .05; **P < .01; ***P < .001

**FIGURE 6:**
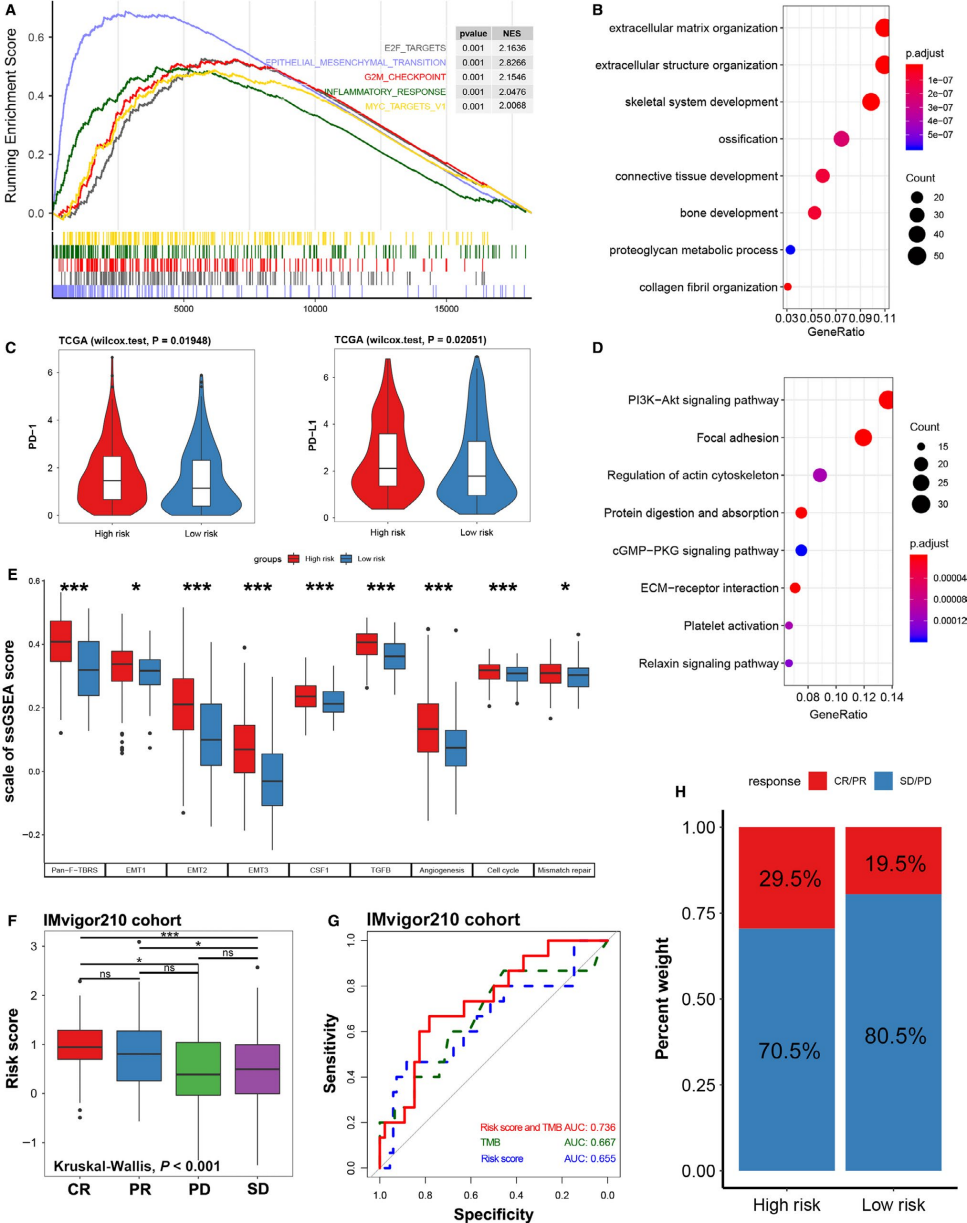
Function annotation, immune checkpoint expression in different risk groups, and immune signature predicts immunotherapy benefit. (A) Enrichment plots of GSEA based on ISS, the blue line shows that the EMT signaling pathway is significantly enriched in high‐risk patients, NES: normalized enrichment score. (B) GO enrichment plot of the top 500 genes with the highest correlation with ISS. (C) Expression of immune checkpoints (PD‐1 and PD‐L1) between high‐ and low‐risk groups. (D) KEGG enrichment plot of the top 500 genes with the highest correlation with ISS. (E) Histogram of standardized ssGSEA scores for signaling pathways between different risk groups. (F) Distribution of ISS in groups with different anti‐PD‐L1 clinical response statuses (CR, complete response; PR, partial response; PD, progressive disease; SD, stable disease). (G) ROC curve used to measure the predictive value of immune signature for immunosuppressive benefits. (H) Rate of clinical response (CR/PR and SD/PD) to immunotherapy in high‐ or low‐risk groups. *P < .05; **P < .01; ***P < .001

By using the “quanTIseq” algorithm, we quantified the absolute abundance of tumor immune cell infiltration in BCa. The results indicate that macrophage (M1 and M2), Tregs, and neutrophil predominate in BCa (Figure 5A); the results also exhibited higher infiltration of macrophage, monocytes, and Tregs, and lower infiltration of neutrophil in the high‐risk group (Figure 5B). Macrophage and Tregs have a strong positive correlation with ISS, while neutrophil has a negative correlation with ISS (Figure S2A). Besides, we found that higher macrophages or Tregs infiltration tended to have a poor prognosis (Figure 5C). With the “estimate” R package based on ssGSEA algorithm, we explored the immune infiltration between high‐ and low‐risk groups from an overall perspective, and found that the high‐risk group had higher immune and stromal infiltration (Figure 5D,E). We explored the expression of immune checkpoints between the high‐ and low‐risk groups and found that the high‐risk group had higher PD‐1, PD‐L1, CTLA4, LAG‐3, and TIM‐3 expressions (Figure 6C, Figure S2C‐E). Through pan‐cancer analysis, we found that immune signature not only had prognostic value in BCa, but also had good predictive efficacy in cervical squamous cell carcinoma and endocervical adenocarcinoma (CESC), head and neck squamous cell carcinoma (HNSC), kidney renal clear cell carcinoma (KIRC), kidney renal papillary cell carcinoma (KIRP), liver hepatocellular carcinoma (LIHC), ovarian serous cystadenocarcinoma (OV), and pan‐cancer (Figure S2F, Table S3).

### 3.5 Functional and pathway annotation for immune signature

The results of GO analysis indicated that the immune signature was related to the biological functions of the extracellular matrix organization, extracellular structure organization, connective tissue development, bone development, etc (Figure 6B, Table S4). The results of KEGG analysis showed that immune signature was mainly enriched in PI3K‐Akt, focal adhesion, and ECM‐receptor interaction‐related signaling pathways (Figure 6D, Table S5). GSEA analysis results exhibited that patients with high ISS mainly had over‐activation of EMT, inflammatory response, and G2M checkpoint signaling pathway (Figure 6A, Figure S2B, Table S6). We performed ssGSEA analysis of immune signature based on published signaling pathway signature (Table S2), and the results showed that EMT, TGF‐β, CSF‐1, etc inhibitory immune signaling pathways were significantly activated in the high‐risk group, the same is true of cell cycle and mismatched repair signaling pathways (Figure 6E). TGF‐β, EMT, angiogenesis these stromal relevant pathways were upregulated in patients with high ISS, which was consistent with the above result (Figure 5E).

### 3.6 Immune signature predicts immunotherapy benefits

Most solid tumors can be classified into three immunological phenotypes: immune‐inflamed, immune‐excluded, or immune desert.^39^, ^40^ To explore whether the immune signature could predict the immunological phenotype of BCa, we studied our immune signature in a large cohort (IMvigor210) of patients with urothelial carcinoma treated with PD‐L1 inhibitors (atezolizumab). In this cohort, 47% of tumor patients present an immune‐excluded phenotype, immune desert, and immune‐inflamed phenotype accounted for 27% and 26% of the total number of patients, respectively. Our results showed that the ISS of the immune‐excluded phenotype was significantly higher than that of the immune desert or the immune‐inflamed phenotype (Figure S3D). The results exhibited that the immune signature showed prognostic value only in patients treated with PD‐L1 and BCG (Figure S3A‐C). And, we further discuss the differences in immunosuppressive benefits between high‐ and low‐risk groups based on immune signature, and it turned out that patients in the high‐risk group had a higher complete response (CR)/partial response (PR) rate than those in the low‐risk group (Figure 6H). Also, the ISS of patients with clinical response of CR was significantly higher than that of patients with progressive disease (PD) or stable disease (SD) (*P* < .05). The ISS of patients with clinical response of PR was also higher than that of SD groups, even though there was no difference with PD groups (*P* < .05, Figure 6F). Moreover, the immune signature combined with TMB could accurately distinguish CR/PR from stable disease (SD)/progressive disease (PD) (AUC = 0.736, Figure 6G).

## 4 DISCUSSION

Through an in‐depth exploration of TCGA BCa data, we developed a classifier with comprehensive predictive value, which could indirectly predict the prognosis and immunotherapy response of BCa through gene expression of 13‐mRNA, thereby to guide more precision immunotherapy. Stratified analysis of BCa immune microenvironment revealed a significant positive correlation between ISS and inhibitory immune cells (Tregs and macrophages M1/M2). However, there was no difference in antitumor immune cells (T cell CD4/CD8) between the high‐risk and low‐risk groups. Moreover, patients with high ISS tend to have immune‐excluded phenotypes in the IMvigor210 cohort.

Tregs are one of the offenders leading to immune escape. It mediates homeostasis peripheral tolerance by inhibiting autoreactive T cells, or secretes cytokines, such as TGF‐β, that restrict T cell infiltration, which usually leads to reduced benefits of immunotherapy and is associated with poor prognosis.^15^, ^41^, ^42^ Tregs, on the other hand, promote tumor progression by promoting angiogenesis.^43^ Our ssGSEA results indicated that patients with a high ISS generally have high activation of TGF‐β and angiogenesis signaling pathways and higher stromal infiltration, which are consistent with the above functions of Tregs. Tumor‐associated macrophages (TAMs) are also among the inhibitory immune cells that contribute to immune escape that can be differentiated into M1 (antitumor) and M2 (pro‐tumor) phenotypes. TAMs tend to acquire M2 carcinogenic phenotypes, which could promote the progress of cancer. Recent studies have found that TAM, like Tregs, can lead to the upregulation of PD‐1 to achieve immune resistance.^44^, ^45^

Our study also found that several vital immune checkpoints were significantly upregulated in patients with high ISS, such as PD‐1, PD‐L1, and CTLA4. These three immune checkpoints have been the focus of research in BCa treatment, and previous studies have found that PD‐L1 can bind to PD‐1 on T cells, B cells, and macrophages activated on the surface of tumor cells, thus showing immunosuppressive effects.^46^, ^47^, ^48^ The high expression level of PD‐L1 has been reported to be significantly correlated with malignancy and poor prognosis of BCa, and such patients have a higher rate of recurrence after surgery.^13^, ^49^, ^50^ Atezolizumab is currently the only FDA‐approved PD‐L1 inhibitor for BCa treatment, yet only a small number of patients benefit from immunotherapy.^51^ Therefore, the immune signature we developed could be stratified according to the sensitivity of BCa patients to PD‐L1 inhibitors.

Our results also indicated that the TGF‐β signaling pathway was significantly activated in patients with high ISS, while the published study has shown that TGF‐β signaling pathway was mostly activated in immune‐excluded or immune‐inflamed phenotypes, which was consistent with our results. The published study suggests that TGF‐β signaling pathway shapes the TME to restrain antitumor immunity by restricting T cell infiltration.^15^ Taken together, our results also provide a potential therapeutic option to suppress tumors, by inhibiting TGF‐β.

After our analysis, we found an interesting phenomenon that patients with high ISS have obvious immune escape phenotypes, so they should tend to have a poor prognosis and poor immune treatment response, but we found through the study of IMvigor210 cohort that patients with high ISS have good immune benefits. This appears to be contradictory, actually otherwise, because our results show that patients with high immune marker scores tend to have high expression of PD‐L1, which makes the immunotherapy response of PD‐L1 inhibitors more effective.

## 5 CONCLUSION

This study constructed an immune marker of BCa, which has a high predictive value in the prognosis and response to immunotherapy of BCa, and can be classified and treated according to the sensitivity of patients to immunotherapy.

## ETHICS APPROVAL AND CONSENT TO PARTICIPATE

Not Applicable.

## CONSENT FOR PUBLICATION

All authors agree to publish.

## AUTHOR CONTRIBUTIONS

YW, LC, and YX conceived and designed the study, YW and LC performed the analysis procedures, YW, LC, LJ, KQ, GW, and YX analyzed the results, YW, LC, MY, YF, and YX contributed to analysis tools, and YW, LC, YX, and XW contributed to the writing of the manuscript. All authors reviewed the manuscript.

## Supporting information

Fig S1‐S3

Table S1‐S6

## Data Availability

RNA‐sequencing (RNA‐seq) and somatic mutation data of the TCGA database can be downloaded from UCSC Xena website (https://gdc.xenahubs.net). All the microarrays data can be downloaded from the Gene Expression Omnibus database (GEO, https://www.ncbi.nlm.nih.gov/geo/). The IMvigor210 cohort was downloaded from the website: http://research‐pub.gene.com/IMvigor210CoreBiologies.
