# Supplementary material for: Immune‐related signature predicts the prognosis and immunotherapy benefit in bladder cancer
Source: Cancer Med. 2020 Aug 25;9(20):7729–41. doi: 10.1002/cam4.3400 (PMC7571842; doi:10.1002/cam4.3400)
Supplement: Supplementary file 1 — Fig S1‐S3 [file CAM4-9-7729-s001.docx]

**Supplementary information**

**Supplementary methods and materials**

The tumor mutation burden (TMB) was calculated as the number of mutations per Mb on the genomic coding region. The screening condition was “PASS” and the mutation frequency was more than 5%. Statistics were performed in R using unpaired Student’s T-test. We calculated the correlation between TMB and ISS, and the results showed no significant difference.

Sources of signatures for ssGSEA analysis

1. Pan-fibroblast TGF-β response signature (Pan-F-TBRS) (1)
2. Epithelial-mesenchymal transition (EMT1) (2)
3. Epithelial-mesenchymal transition (EMT2) (3)
4. Epithelial-mesenchymal transition (EMT3) (4)
5. Angiogenesis (5)
6. Cell cycle (KEGG hsa4110)
7. Mismatch repair (KEGG hsa3430)
8. Colony - stimulating factor 1 (CSF1) (6)

**Supplementary figures**

**
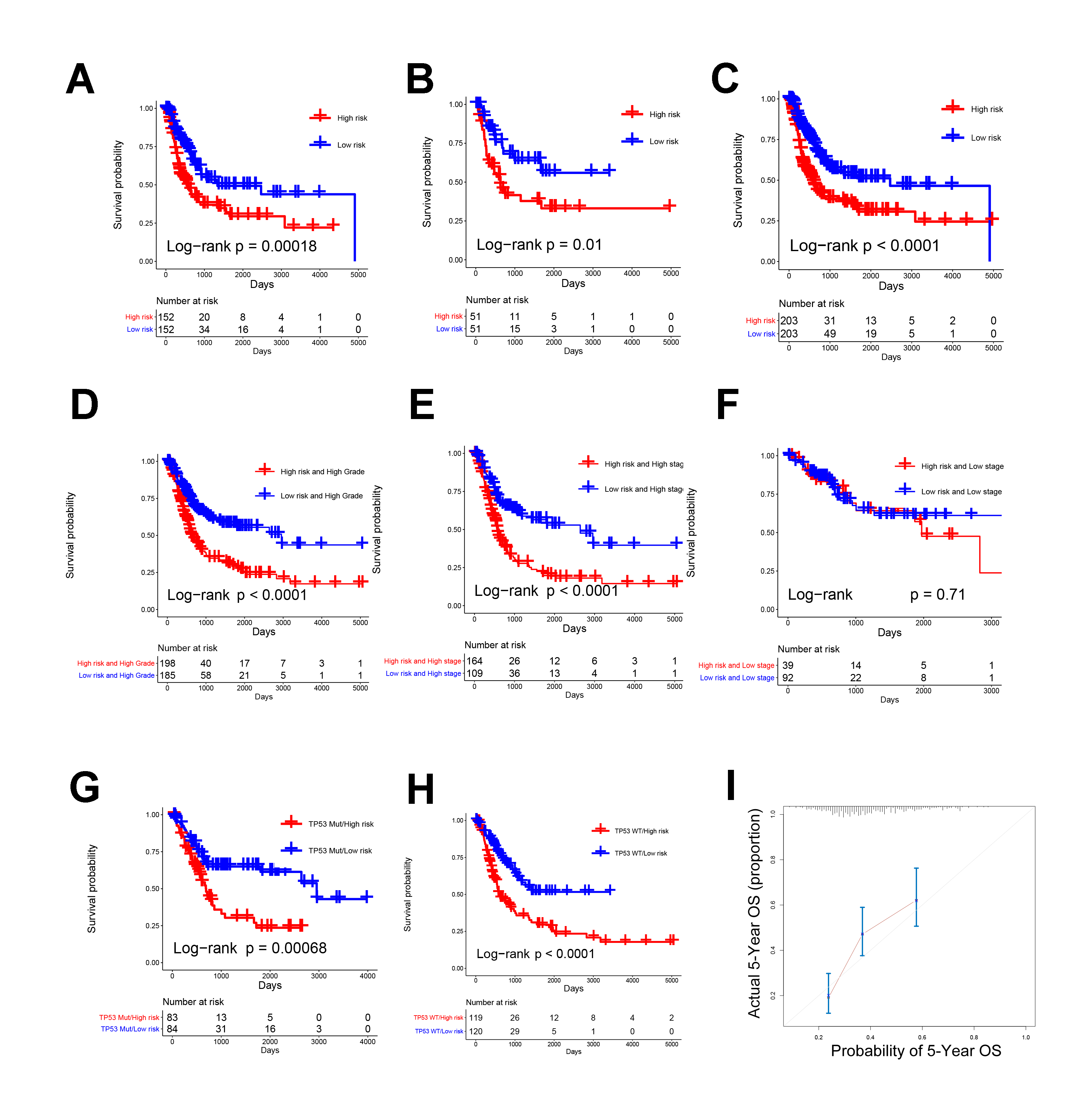
**

**Figure S1. (A-C)** PFS analysis of immune signature in TCGA cohort. **(D-F)** Immune signature were analyzed in subgroups of different stages or grades. **(G-H)** Immune signature were analyzed in subgroups of different TP53 mutation states. **(I)** Plots depict the calibration of the nomogram between predicted and observed 5-year outcomes.


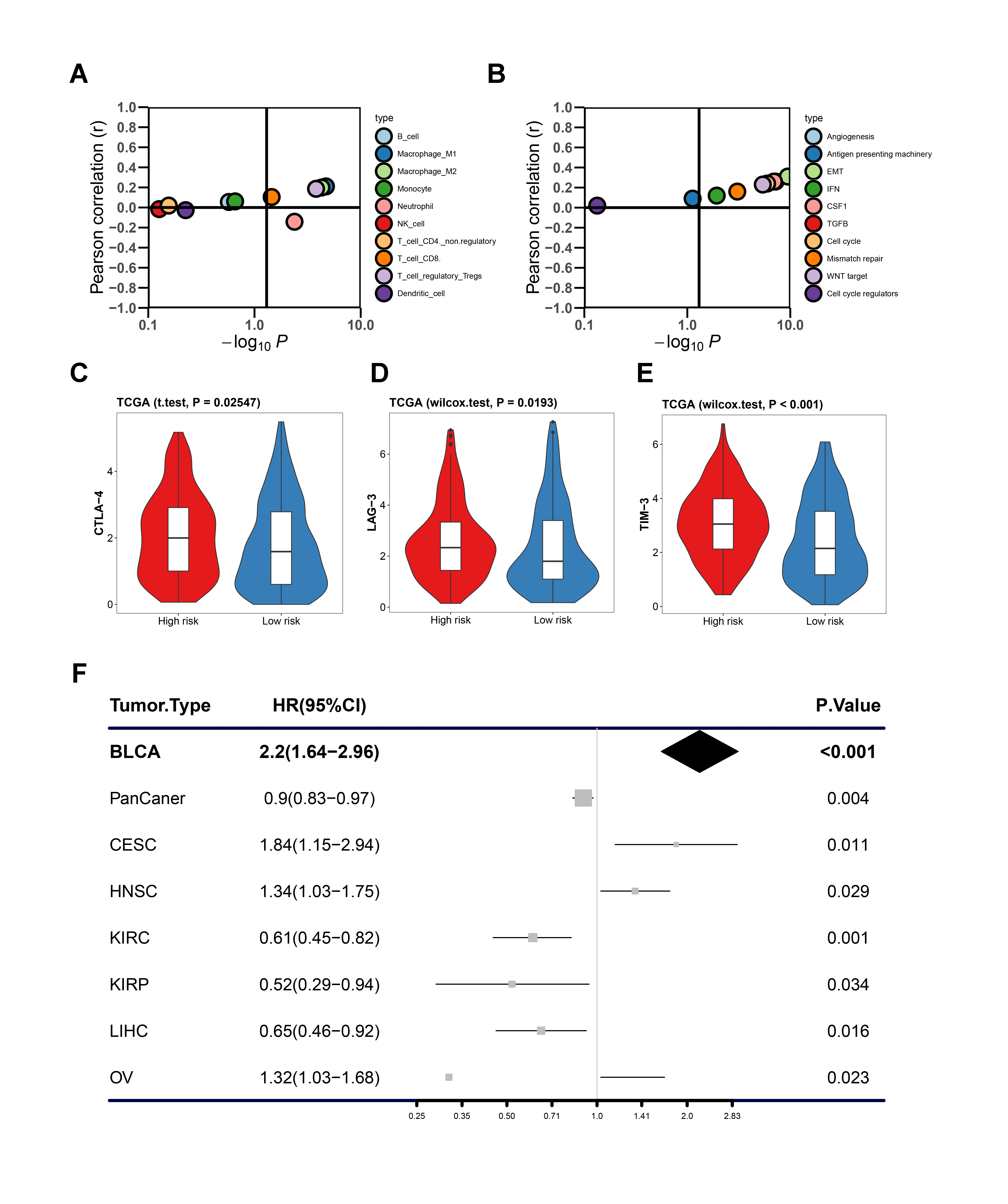


**Figure S2.** Correlation analysis of ISS with SSGSEA fraction of immune cell infiltration **(A)** and immune related pathways **(B).** Expression of CTLA-4 **(C),** LAG-3 **(D),** TIM-3 **(E)** between the high- and low-risk group. **(F)** Pan-cancer analysis for immune signature.


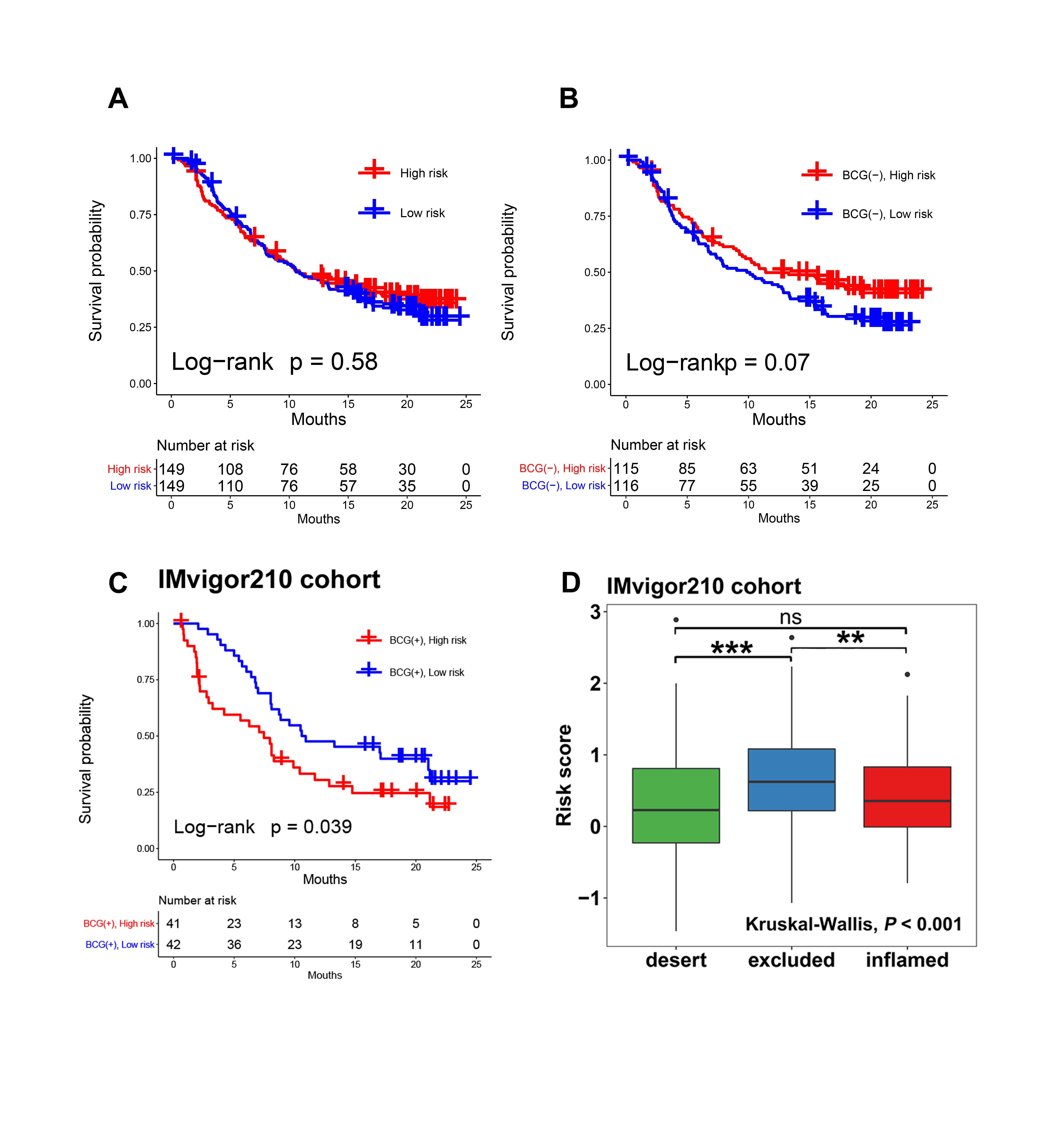


**Figure S3. (A-C)** Subgroup survival analysis of immune signature in the IMvigor210 cohort. **(D)** Box plots of ISS in different immune phenotypes.

# References

1. Mariathasan S, Turley SJ, Nickles D, Castiglioni A, Yuen K, Wang Y, et al. TGFbeta attenuates tumour response to PD-L1 blockade by contributing to exclusion of T cells. Nature. 2018;554(7693):544-8.

2. Damrauer JS, Hoadley KA, Chism DD, Fan C, Tiganelli CJ, Wobker SE, et al. Intrinsic subtypes of high-grade bladder cancer reflect the hallmarks of breast cancer biology. Proc Natl Acad Sci U S A. 2014;111(8):3110-5.

3. Hugo W, Zaretsky JM, Sun L, Song C, Moreno BH, Hu-Lieskovan S, et al. Genomic and Transcriptomic Features of Response to Anti-PD-1 Therapy in Metastatic Melanoma. Cell. 2016;165(1):35-44.

4. Hedegaard J, Lamy P, Nordentoft I, Algaba F, Hoyer S, Ulhoi BP, et al. Comprehensive Transcriptional Analysis of Early-Stage Urothelial Carcinoma. Cancer Cell. 2016;30(1):27-42.

5. Sjodahl G, Lauss M, Lovgren K, Chebil G, Gudjonsson S, Veerla S, et al. A molecular taxonomy for urothelial carcinoma. Clin Cancer Res. 2012;18(12):3377-86.

6. Beck AH, Espinosa I, Edris B, Li R, Montgomery K, Zhu S, et al. The macrophage colony-stimulating factor 1 response signature in breast carcinoma. Clin Cancer Res. 2009;15(3):778-87.
