# Supplementary material for: Immune‐related signature predicts the prognosis and immunotherapy benefit in bladder cancer
Source: Cancer Med. 2020 Aug 25;9(20):7729–41. doi: 10.1002/cam4.3400 (PMC7571842; doi:10.1002/cam4.3400)
Supplement: Supplementary file 2 — Table S1‐S6 [file CAM4-9-7729-s002.docx]

| **Table S1.** Details of the prognosis signature. | |
| --- | --- |
| **GeneName** | **coef** |
| SULT1C2 | -0.285729313 |
| EGFL6 | -0.132834959 |
| DUSP2 | -0.047080965 |
| BLNK | -0.036593582 |
| BATF | -0.027054952 |
| PPFIBP2 | -0.015812131 |
| HES1 | -0.00830084 |
| THBS4 | 0.011476589 |
| CPA3 | 0.061002869 |
| NEFL | 0.069959198 |
| VWF | 0.0806877 |
| LRP8 | 0.09542848 |
| PDGFD | 0.110454703 |

**Table S2.** Published signatures used in ssGSEA analysis.

| Gene Signature | Source |
| --- | --- |
| EMT-1 | PMID: 24520177 |
| EMT2 | PMID: 26997480 |
| EMT3 | PMID: 27321955 |
| Pan-F-TBRS | PMID: 29443960 |
| Angiogenesis | PMID: 22553347 |
| Cell cycle | KEGG hsa4110 |
| Mismatch repair | KEGG hsa3430 |
| CSF-1 | PMID: 19188147 |

| **Table S3.** Pan-cancer analysis of immune signature. | | |
| --- | --- | --- |
| **Tumor Type** | **HR(95%CI)** | **P.Value** |
| BLCA | 2.20(1.64-2.96) | <0.001 |
| KIRC | 0.61(0.45-0.82) | 0.001 |
| PanCaner | 0.9(0.83-0.97) | 0.004 |
| CESC | 1.84(1.15-2.94) | 0.011 |
| LIHC | 0.65(0.46-0.92) | 0.016 |
| OV | 1.32(1.03-1.68) | 0.023 |
| HNSC | 1.34(1.03-1.75) | 0.029 |
| KIRP | 0.52(0.29-0.94) | 0.034 |
| PCPG | 5.46(1.1-27.09) | 0.08 |
| LUAD | 1.27(0.95-1.7) | 0.106 |
| LGG | 0.78(0.55-1.11) | 0.167 |
| UCS | 0.63(0.32-1.22) | 0.168 |
| READ | 1.98(0.74-5.29) | 0.175 |
| PAAD | 0.79(0.53-1.19) | 0.247 |
| CHOL | 1.64(0.65-4.17) | 0.276 |
| THCA | 1.67(0.63-4.45) | 0.314 |
| GBM | 0.84(0.57-1.23) | 0.362 |
| UCEC | 0.73(0.37-1.45) | 0.364 |
| KICH | 0.56(0.15-2.07) | 0.399 |
| ESCA | 0.83(0.53-1.3) | 0.402 |
| COAD | 0.82(0.51-1.32) | 0.413 |
| DLBC | 1.65(0.44-6.17) | 0.422 |
| SARC | 0.9(0.6-1.34) | 0.611 |
| SKCM | 0.84(0.4-1.78) | 0.624 |
| BRCA | 1.07(0.78-1.47) | 0.66 |
| UVM | 1.17(0.5-2.73) | 0.714 |
| MESO | 1.08(0.68-1.71) | 0.734 |
| LUSC | 1.04(0.79-1.36) | 0.772 |
| TGCT | 0.78(0.11-5.62) | 0.797 |
| PRAD | 0.87(0.25-3.04) | 0.819 |
| THYM | 1.14(0.28-4.57) | 0.856 |
| ACC | 0.96(0.45-2.04) | 0.921 |
| STAD | 0.99(0.72-1.35) | 0.957 |

| **Table S4.** Results of immune signature GO analysis. | | |  |  |  |
| --- | --- | --- | --- | --- | --- |
| **ONTOLOGY** | **ID** | **Description** | **GeneRatio** | **pvalue** | **Count** |
| BP | GO:0030198 | extracellular matrix organization | 50/456 | <0.001 | 50 |
| BP | GO:0043062 | extracellular structure organization | 50/456 | <0.001 | 50 |
| BP | GO:0001501 | skeletal system development | 45/456 | <0.001 | 45 |
| BP | GO:0030199 | collagen fibril organization | 14/456 | <0.001 | 14 |
| BP | GO:0060348 | bone development | 24/456 | <0.001 | 24 |
| BP | GO:0061448 | connective tissue development | 27/456 | <0.001 | 27 |
| BP | GO:0001503 | ossification | 34/456 | <0.001 | 34 |
| BP | GO:0006029 | proteoglycan metabolic process | 15/456 | <0.001 | 15 |
| BP | GO:0051216 | cartilage development | 22/456 | <0.001 | 22 |
| BP | GO:0006024 | glycosaminoglycan biosynthetic process | 17/456 | <0.001 | 17 |
| BP | GO:0006023 | aminoglycan biosynthetic process | 17/456 | <0.001 | 17 |
| BP | GO:0030203 | glycosaminoglycan metabolic process | 20/456 | <0.001 | 20 |
| BP | GO:0001667 | ameboidal-type cell migration | 35/456 | <0.001 | 35 |
| BP | GO:0031589 | cell-substrate adhesion | 29/456 | <0.001 | 29 |
| BP | GO:0006022 | aminoglycan metabolic process | 20/456 | <0.001 | 20 |
| BP | GO:0007160 | cell-matrix adhesion | 23/456 | <0.001 | 23 |
| BP | GO:0002062 | chondrocyte differentiation | 15/456 | <0.001 | 15 |
| BP | GO:0010975 | regulation of neuron projection development | 34/456 | <0.001 | 34 |
| BP | GO:0050650 | chondroitin sulfate proteoglycan biosynthetic process | 9/456 | <0.001 | 9 |
| BP | GO:0002576 | platelet degranulation | 17/456 | <0.001 | 17 |
| BP | GO:0060840 | artery development | 15/456 | <0.001 | 15 |
| BP | GO:0048762 | mesenchymal cell differentiation | 21/456 | <0.001 | 21 |
| BP | GO:0060485 | mesenchyme development | 24/456 | <0.001 | 24 |
| BP | GO:0006936 | muscle contraction | 28/456 | <0.001 | 28 |
| BP | GO:0050878 | regulation of body fluid levels | 35/456 | <0.001 | 35 |
| BP | GO:0050654 | chondroitin sulfate proteoglycan metabolic process | 10/456 | <0.001 | 10 |
| BP | GO:0030166 | proteoglycan biosynthetic process | 11/456 | <0.001 | 11 |
| BP | GO:0048483 | autonomic nervous system development | 10/456 | <0.001 | 10 |
| BP | GO:0010631 | epithelial cell migration | 28/456 | <0.001 | 28 |
| BP | GO:0090132 | epithelium migration | 28/456 | <0.001 | 28 |
| BP | GO:1903510 | mucopolysaccharide metabolic process | 15/456 | <0.001 | 15 |
| BP | GO:0035904 | aorta development | 11/456 | <0.001 | 11 |
| BP | GO:0030206 | chondroitin sulfate biosynthetic process | 8/456 | <0.001 | 8 |
| BP | GO:0051057 | positive regulation of small GTPase mediated signal transduction | 11/456 | <0.001 | 11 |
| BP | GO:0090130 | tissue migration | 28/456 | <0.001 | 28 |
| BP | GO:0018108 | peptidyl-tyrosine phosphorylation | 27/456 | <0.001 | 27 |
| BP | GO:0018212 | peptidyl-tyrosine modification | 27/456 | <0.001 | 27 |
| BP | GO:0001764 | neuron migration | 17/456 | <0.001 | 17 |
| BP | GO:0030168 | platelet activation | 17/456 | <0.001 | 17 |
| BP | GO:0007264 | small GTPase mediated signal transduction | 32/456 | <0.001 | 32 |
| BP | GO:0030204 | chondroitin sulfate metabolic process | 9/456 | <0.001 | 9 |
| BP | GO:0001763 | morphogenesis of a branching structure | 19/456 | <0.001 | 19 |
| BP | GO:0003012 | muscle system process | 31/456 | <0.001 | 31 |
| BP | GO:0050673 | epithelial cell proliferation | 30/456 | <0.001 | 30 |
| BP | GO:0046579 | positive regulation of Ras protein signal transduction | 10/456 | <0.001 | 10 |
| BP | GO:0007599 | hemostasis | 26/456 | <0.001 | 26 |
| BP | GO:0060560 | developmental growth involved in morphogenesis | 20/456 | <0.001 | 20 |
| BP | GO:0061138 | morphogenesis of a branching epithelium | 18/456 | <0.001 | 18 |
| BP | GO:0034330 | cell junction organization | 23/456 | <0.001 | 23 |
| BP | GO:0001935 | endothelial cell proliferation | 18/456 | <0.001 | 18 |
| BP | GO:0048839 | inner ear development | 18/456 | <0.001 | 18 |
| BP | GO:0007596 | blood coagulation | 25/456 | <0.001 | 25 |
| BP | GO:0022612 | gland morphogenesis | 14/456 | <0.001 | 14 |
| BP | GO:0045666 | positive regulation of neuron differentiation | 26/456 | <0.001 | 26 |
| BP | GO:0001704 | formation of primary germ layer | 14/456 | <0.001 | 14 |
| BP | GO:0001936 | regulation of endothelial cell proliferation | 17/456 | <0.001 | 17 |
| BP | GO:0048675 | axon extension | 13/456 | <0.001 | 13 |
| BP | GO:0032963 | collagen metabolic process | 13/456 | <0.001 | 13 |
| BP | GO:0050817 | coagulation | 25/456 | <0.001 | 25 |
| BP | GO:0050772 | positive regulation of axonogenesis | 11/456 | <0.001 | 11 |
| BP | GO:0010632 | regulation of epithelial cell migration | 23/456 | <0.001 | 23 |
| BP | GO:0051056 | regulation of small GTPase mediated signal transduction | 24/456 | <0.001 | 24 |
| BP | GO:0050769 | positive regulation of neurogenesis | 30/456 | <0.001 | 30 |
| BP | GO:0071560 | cellular response to transforming growth factor beta stimulus | 20/456 | <0.001 | 20 |
| BP | GO:0034329 | cell junction assembly | 19/456 | <0.001 | 19 |
| BP | GO:0061564 | axon development | 29/456 | <0.001 | 29 |
| BP | GO:0003170 | heart valve development | 10/456 | <0.001 | 10 |
| BP | GO:0050678 | regulation of epithelial cell proliferation | 26/456 | <0.001 | 26 |
| BP | GO:0048844 | artery morphogenesis | 11/456 | <0.001 | 11 |
| BP | GO:0071559 | response to transforming growth factor beta | 20/456 | <0.001 | 20 |
| BP | GO:0090066 | regulation of anatomical structure size | 29/456 | <0.001 | 29 |
| BP | GO:0014031 | mesenchymal cell development | 10/456 | <0.001 | 10 |
| BP | GO:0003179 | heart valve morphogenesis | 9/456 | <0.001 | 9 |
| BP | GO:1990138 | neuron projection extension | 15/456 | <0.001 | 15 |
| BP | GO:0072001 | renal system development | 22/456 | <0.001 | 22 |
| BP | GO:0031346 | positive regulation of cell projection organization | 25/456 | <0.001 | 25 |
| BP | GO:0043583 | ear development | 18/456 | <0.001 | 18 |
| BP | GO:0001822 | kidney development | 21/456 | <0.001 | 21 |
| BP | GO:0007409 | axonogenesis | 26/456 | <0.001 | 26 |
| BP | GO:0001655 | urogenital system development | 23/456 | <0.001 | 23 |
| BP | GO:0007265 | Ras protein signal transduction | 23/456 | <0.001 | 23 |
| BP | GO:0014033 | neural crest cell differentiation | 10/456 | <0.001 | 10 |
| BP | GO:0010770 | positive regulation of cell morphogenesis involved in differentiation | 14/456 | <0.001 | 14 |
| BP | GO:0045165 | cell fate commitment | 19/456 | <0.001 | 19 |
| BP | GO:0010769 | regulation of cell morphogenesis involved in differentiation | 20/456 | <0.001 | 20 |
| BP | GO:0007369 | gastrulation | 16/456 | <0.001 | 16 |
| BP | GO:0048667 | cell morphogenesis involved in neuron differentiation | 30/456 | <0.001 | 30 |
| BP | GO:0030318 | melanocyte differentiation | 6/456 | <0.001 | 6 |
| BP | GO:0035987 | endodermal cell differentiation | 8/456 | <0.001 | 8 |
| BP | GO:0010976 | positive regulation of neuron projection development | 20/456 | <0.001 | 20 |
| BP | GO:0098751 | bone cell development | 7/456 | <0.001 | 7 |
| BP | GO:0001649 | osteoblast differentiation | 18/456 | <0.001 | 18 |
| BP | GO:0022604 | regulation of cell morphogenesis | 27/456 | <0.001 | 27 |
| BP | GO:0001938 | positive regulation of endothelial cell proliferation | 12/456 | <0.001 | 12 |
| BP | GO:0060562 | epithelial tube morphogenesis | 22/456 | <0.001 | 22 |
| BP | GO:0007009 | plasma membrane organization | 11/456 | <0.001 | 11 |
| BP | GO:0007435 | salivary gland morphogenesis | 7/456 | <0.001 | 7 |
| BP | GO:0035909 | aorta morphogenesis | 7/456 | <0.001 | 7 |
| BP | GO:0030516 | regulation of axon extension | 10/456 | <0.001 | 10 |
| BP | GO:0048754 | branching morphogenesis of an epithelial tube | 14/456 | <0.001 | 14 |
| BP | GO:0014032 | neural crest cell development | 9/456 | <0.001 | 9 |
| BP | GO:0032835 | glomerulus development | 9/456 | <0.001 | 9 |
| BP | GO:0050770 | regulation of axonogenesis | 14/456 | <0.001 | 14 |
| BP | GO:0021987 | cerebral cortex development | 12/456 | <0.001 | 12 |
| BP | GO:0003205 | cardiac chamber development | 15/456 | <0.001 | 15 |
| BP | GO:0060384 | innervation | 6/456 | <0.001 | 6 |
| BP | GO:0003279 | cardiac septum development | 12/456 | <0.001 | 12 |
| BP | GO:0007044 | cell-substrate junction assembly | 11/456 | <0.001 | 11 |
| BP | GO:0048565 | digestive tract development | 13/456 | <0.001 | 13 |
| BP | GO:0001706 | endoderm formation | 8/456 | <0.001 | 8 |
| BP | GO:0001755 | neural crest cell migration | 7/456 | <0.001 | 7 |
| BP | GO:0048864 | stem cell development | 9/456 | <0.001 | 9 |
| BP | GO:0046578 | regulation of Ras protein signal transduction | 17/456 | <0.001 | 17 |
| BP | GO:0045992 | negative regulation of embryonic development | 6/456 | <0.001 | 6 |
| BP | GO:0008361 | regulation of cell size | 14/456 | <0.001 | 14 |
| BP | GO:0007517 | muscle organ development | 25/456 | <0.001 | 25 |
| BP | GO:0007431 | salivary gland development | 7/456 | <0.001 | 7 |
| BP | GO:0002063 | chondrocyte development | 6/456 | <0.001 | 6 |
| BP | GO:0021783 | preganglionic parasympathetic fiber development | 5/456 | <0.001 | 5 |
| BP | GO:0061298 | retina vasculature development in camera-type eye | 5/456 | <0.001 | 5 |
| BP | GO:0030278 | regulation of ossification | 16/456 | <0.001 | 16 |
| BP | GO:0032964 | collagen biosynthetic process | 8/456 | <0.001 | 8 |
| BP | GO:0021537 | telencephalon development | 18/456 | <0.001 | 18 |
| BP | GO:0034446 | substrate adhesion-dependent cell spreading | 10/456 | <0.001 | 10 |
| BP | GO:0016049 | cell growth | 27/456 | <0.001 | 27 |
| BP | GO:0072006 | nephron development | 13/456 | <0.001 | 13 |
| BP | GO:0045773 | positive regulation of axon extension | 7/456 | <0.001 | 7 |
| BP | GO:0001885 | endothelial cell development | 8/456 | <0.001 | 8 |
| BP | GO:0031102 | neuron projection regeneration | 8/456 | <0.001 | 8 |
| BP | GO:0003007 | heart morphogenesis | 18/456 | <0.001 | 18 |
| BP | GO:0006939 | smooth muscle contraction | 11/456 | <0.001 | 11 |
| BP | GO:0045446 | endothelial cell differentiation | 11/456 | <0.001 | 11 |
| BP | GO:0055123 | digestive system development | 13/456 | <0.001 | 13 |
| BP | GO:0006929 | substrate-dependent cell migration | 6/456 | <0.001 | 6 |
| BP | GO:0001886 | endothelial cell morphogenesis | 4/456 | <0.001 | 4 |
| BP | GO:0061299 | retina vasculature morphogenesis in camera-type eye | 4/456 | <0.001 | 4 |
| BP | GO:0003158 | endothelium development | 12/456 | <0.001 | 12 |
| BP | GO:0051493 | regulation of cytoskeleton organization | 27/456 | <0.001 | 27 |
| BP | GO:0090287 | regulation of cellular response to growth factor stimulus | 19/456 | <0.001 | 19 |
| BP | GO:0048486 | parasympathetic nervous system development | 5/456 | <0.001 | 5 |
| BP | GO:0021543 | pallium development | 14/456 | <0.001 | 14 |
| BP | GO:0061387 | regulation of extent of cell growth | 10/456 | <0.001 | 10 |
| BP | GO:0045995 | regulation of embryonic development | 12/456 | <0.001 | 12 |
| BP | GO:0021675 | nerve development | 9/456 | <0.001 | 9 |
| BP | GO:0003206 | cardiac chamber morphogenesis | 12/456 | <0.001 | 12 |
| BP | GO:0048846 | axon extension involved in axon guidance | 5/456 | <0.001 | 5 |
| BP | GO:1902284 | neuron projection extension involved in neuron projection guidance | 5/456 | <0.001 | 5 |
| BP | GO:0021561 | facial nerve development | 4/456 | <0.001 | 4 |
| BP | GO:0021604 | cranial nerve structural organization | 4/456 | <0.001 | 4 |
| BP | GO:0021610 | facial nerve morphogenesis | 4/456 | <0.001 | 4 |
| BP | GO:0021954 | central nervous system neuron development | 9/456 | <0.001 | 9 |
| BP | GO:0050931 | pigment cell differentiation | 6/456 | <0.001 | 6 |
| BP | GO:0045765 | regulation of angiogenesis | 23/456 | <0.001 | 23 |
| BP | GO:0048588 | developmental cell growth | 16/456 | <0.001 | 16 |
| BP | GO:0045778 | positive regulation of ossification | 10/456 | <0.001 | 10 |
| BP | GO:0010634 | positive regulation of epithelial cell migration | 14/456 | <0.001 | 14 |
| BP | GO:0050900 | leukocyte migration | 26/456 | <0.001 | 26 |
| BP | GO:0006816 | calcium ion transport | 24/456 | <0.001 | 24 |
| BP | GO:0032535 | regulation of cellular component size | 20/456 | <0.001 | 20 |
| BP | GO:0051271 | negative regulation of cellular component movement | 22/456 | <0.001 | 22 |
| BP | GO:0051924 | regulation of calcium ion transport | 17/456 | <0.001 | 17 |
| BP | GO:0002064 | epithelial cell development | 15/456 | <0.001 | 15 |
| BP | GO:0032970 | regulation of actin filament-based process | 21/456 | <0.001 | 21 |
| BP | GO:0060537 | muscle tissue development | 23/456 | <0.001 | 23 |
| BP | GO:0016055 | Wnt signaling pathway | 27/456 | <0.001 | 27 |
| BP | GO:0071230 | cellular response to amino acid stimulus | 8/456 | <0.001 | 8 |
| BP | GO:0198738 | cell-cell signaling by wnt | 27/456 | <0.001 | 27 |
| BP | GO:0055001 | muscle cell development | 13/456 | <0.001 | 13 |
| BP | GO:0035272 | exocrine system development | 7/456 | <0.001 | 7 |
| BP | GO:0090102 | cochlea development | 7/456 | <0.001 | 7 |
| BP | GO:0030900 | forebrain development | 22/456 | <0.001 | 22 |
| BP | GO:1905330 | regulation of morphogenesis of an epithelium | 14/456 | <0.001 | 14 |
| BP | GO:0051928 | positive regulation of calcium ion transport | 11/456 | <0.001 | 11 |
| BP | GO:0032956 | regulation of actin cytoskeleton organization | 19/456 | <0.001 | 19 |
| BP | GO:0048705 | skeletal system morphogenesis | 15/456 | <0.001 | 15 |
| BP | GO:0060445 | branching involved in salivary gland morphogenesis | 5/456 | <0.001 | 5 |
| BP | GO:0070208 | protein heterotrimerization | 4/456 | <0.001 | 4 |
| BP | GO:0043542 | endothelial cell migration | 18/456 | <0.001 | 18 |
| BP | GO:0010718 | positive regulation of epithelial to mesenchymal transition | 7/456 | <0.001 | 7 |
| BP | GO:0009187 | cyclic nucleotide metabolic process | 6/456 | <0.001 | 6 |
| BP | GO:0043114 | regulation of vascular permeability | 6/456 | <0.001 | 6 |
| BP | GO:0070838 | divalent metal ion transport | 25/456 | <0.001 | 25 |
| BP | GO:0045669 | positive regulation of osteoblast differentiation | 8/456 | <0.001 | 8 |
| BP | GO:0021952 | central nervous system projection neuron axonogenesis | 5/456 | <0.001 | 5 |
| BP | GO:0072511 | divalent inorganic cation transport | 25/456 | <0.001 | 25 |
| BP | GO:1905314 | semi-lunar valve development | 6/456 | <0.001 | 6 |
| BP | GO:0001101 | response to acid chemical | 20/456 | <0.001 | 20 |
| BP | GO:0046068 | cGMP metabolic process | 4/456 | <0.001 | 4 |
| BP | GO:0044272 | sulfur compound biosynthetic process | 14/456 | <0.001 | 14 |
| BP | GO:0003015 | heart process | 18/456 | <0.001 | 18 |
| BP | GO:0048863 | stem cell differentiation | 16/456 | <0.001 | 16 |
| BP | GO:0030324 | lung development | 13/456 | <0.001 | 13 |
| BP | GO:0048568 | embryonic organ development | 23/456 | <0.001 | 23 |
| BP | GO:0045785 | positive regulation of cell adhesion | 22/456 | <0.001 | 22 |
| BP | GO:0017145 | stem cell division | 6/456 | <0.001 | 6 |
| BP | GO:0046834 | lipid phosphorylation | 6/456 | <0.001 | 6 |
| BP | GO:0060541 | respiratory system development | 14/456 | <0.001 | 14 |
| BP | GO:0030201 | heparan sulfate proteoglycan metabolic process | 5/456 | <0.001 | 5 |
| BP | GO:0061437 | renal system vasculature development | 5/456 | <0.001 | 5 |
| BP | GO:0061440 | kidney vasculature development | 5/456 | <0.001 | 5 |
| BP | GO:0050651 | dermatan sulfate proteoglycan biosynthetic process | 4/456 | <0.001 | 4 |
| BP | GO:0072160 | nephron tubule epithelial cell differentiation | 4/456 | <0.001 | 4 |
| BP | GO:0031099 | regeneration | 14/456 | <0.001 | 14 |
| BP | GO:0060349 | bone morphogenesis | 9/456 | <0.001 | 9 |
| BP | GO:1901888 | regulation of cell junction assembly | 9/456 | <0.001 | 9 |
| BP | GO:0030111 | regulation of Wnt signaling pathway | 20/456 | <0.001 | 20 |
| BP | GO:0030323 | respiratory tube development | 13/456 | <0.001 | 13 |
| BP | GO:0014909 | smooth muscle cell migration | 9/456 | 0.001 | 9 |
| BP | GO:0050808 | synapse organization | 22/456 | 0.001 | 22 |
| BP | GO:0086002 | cardiac muscle cell action potential involved in contraction | 7/456 | 0.001 | 7 |
| BP | GO:1903522 | regulation of blood circulation | 18/456 | 0.001 | 18 |
| BP | GO:0007492 | endoderm development | 8/456 | 0.001 | 8 |
| BP | GO:0001958 | endochondral ossification | 5/456 | 0.001 | 5 |
| BP | GO:0036075 | replacement ossification | 5/456 | 0.001 | 5 |
| BP | GO:0032872 | regulation of stress-activated MAPK cascade | 15/456 | 0.001 | 15 |
| BP | GO:0001558 | regulation of cell growth | 22/456 | 0.001 | 22 |
| BP | GO:0001709 | cell fate determination | 6/456 | 0.001 | 6 |
| BP | GO:0014002 | astrocyte development | 6/456 | 0.001 | 6 |
| BP | GO:0048066 | developmental pigmentation | 6/456 | 0.001 | 6 |
| BP | GO:0030048 | actin filament-based movement | 11/456 | 0.001 | 11 |
| BP | GO:0050730 | regulation of peptidyl-tyrosine phosphorylation | 16/456 | 0.001 | 16 |
| BP | GO:0030193 | regulation of blood coagulation | 8/456 | 0.001 | 8 |
| BP | GO:0022029 | telencephalon cell migration | 7/456 | 0.001 | 7 |
| BP | GO:0070302 | regulation of stress-activated protein kinase signaling cascade | 15/456 | 0.001 | 15 |
| BP | GO:0031214 | biomineral tissue development | 12/456 | 0.001 | 12 |
| BP | GO:0008090 | retrograde axonal transport | 4/456 | 0.001 | 4 |
| BP | GO:0036035 | osteoclast development | 4/456 | 0.001 | 4 |
| BP | GO:0050655 | dermatan sulfate proteoglycan metabolic process | 4/456 | 0.001 | 4 |
| BP | GO:0071229 | cellular response to acid chemical | 14/456 | 0.001 | 14 |
| BP | GO:1901342 | regulation of vasculature development | 23/456 | 0.001 | 23 |
| BP | GO:0051403 | stress-activated MAPK cascade | 17/456 | 0.001 | 17 |
| BP | GO:2000146 | negative regulation of cell motility | 20/456 | 0.001 | 20 |
| BP | GO:0021953 | central nervous system neuron differentiation | 13/456 | 0.001 | 13 |
| BP | GO:1900046 | regulation of hemostasis | 8/456 | 0.001 | 8 |
| BP | GO:0043405 | regulation of MAP kinase activity | 19/456 | 0.001 | 19 |
| BP | GO:0003180 | aortic valve morphogenesis | 5/456 | 0.001 | 5 |
| BP | GO:0010863 | positive regulation of phospholipase C activity | 6/456 | 0.001 | 6 |
| BP | GO:0051489 | regulation of filopodium assembly | 6/456 | 0.001 | 6 |
| BP | GO:0070527 | platelet aggregation | 7/456 | 0.001 | 7 |
| BP | GO:0007229 | integrin-mediated signaling pathway | 9/456 | 0.001 | 9 |
| BP | GO:0007178 | transmembrane receptor protein serine/threonine kinase signaling pathway | 19/456 | 0.001 | 19 |
| BP | GO:0010810 | regulation of cell-substrate adhesion | 14/456 | 0.001 | 14 |
| BP | GO:0090288 | negative regulation of cellular response to growth factor stimulus | 12/456 | 0.001 | 12 |
| BP | GO:0008016 | regulation of heart contraction | 16/456 | 0.001 | 16 |
| BP | GO:0010977 | negative regulation of neuron projection development | 11/456 | 0.001 | 11 |
| BP | GO:0014706 | striated muscle tissue development | 21/456 | 0.001 | 21 |
| BP | GO:0003281 | ventricular septum development | 8/456 | 0.001 | 8 |
| BP | GO:0061326 | renal tubule development | 9/456 | 0.001 | 9 |
| BP | GO:2000027 | regulation of animal organ morphogenesis | 16/456 | 0.001 | 16 |
| BP | GO:0040013 | negative regulation of locomotion | 21/456 | 0.001 | 21 |
| BP | GO:0048880 | sensory system development | 20/456 | 0.001 | 20 |
| BP | GO:0060047 | heart contraction | 17/456 | 0.001 | 17 |
| BP | GO:0010470 | regulation of gastrulation | 6/456 | 0.001 | 6 |
| BP | GO:0048708 | astrocyte differentiation | 8/456 | 0.001 | 8 |
| BP | GO:0003184 | pulmonary valve morphogenesis | 4/456 | 0.001 | 4 |
| BP | GO:0048532 | anatomical structure arrangement | 4/456 | 0.001 | 4 |
| BP | GO:0046928 | regulation of neurotransmitter secretion | 9/456 | 0.001 | 9 |
| BP | GO:0021885 | forebrain cell migration | 7/456 | 0.001 | 7 |
| BP | GO:0031098 | stress-activated protein kinase signaling cascade | 17/456 | 0.001 | 17 |
| BP | GO:0021795 | cerebral cortex cell migration | 6/456 | 0.001 | 6 |
| BP | GO:1900274 | regulation of phospholipase C activity | 6/456 | 0.001 | 6 |
| BP | GO:0003018 | vascular process in circulatory system | 12/456 | 0.001 | 12 |
| BP | GO:0007179 | transforming growth factor beta receptor signaling pathway | 13/456 | 0.001 | 13 |
| BP | GO:0050818 | regulation of coagulation | 8/456 | 0.001 | 8 |
| BP | GO:0030336 | negative regulation of cell migration | 19/456 | 0.001 | 19 |
| BP | GO:0072073 | kidney epithelium development | 11/456 | 0.001 | 11 |
| BP | GO:0007274 | neuromuscular synaptic transmission | 4/456 | 0.001 | 4 |
| BP | GO:0050886 | endocrine process | 8/456 | 0.001 | 8 |
| BP | GO:0048017 | inositol lipid-mediated signaling | 12/456 | 0.001 | 12 |
| BP | GO:0003382 | epithelial cell morphogenesis | 5/456 | 0.001 | 5 |
| BP | GO:0007045 | cell-substrate adherens junction assembly | 8/456 | 0.001 | 8 |
| BP | GO:0048041 | focal adhesion assembly | 8/456 | 0.001 | 8 |
| BP | GO:0042063 | gliogenesis | 17/456 | 0.001 | 17 |
| BP | GO:0010959 | regulation of metal ion transport | 20/456 | 0.001 | 20 |
| BP | GO:1902903 | regulation of supramolecular fiber organization | 18/456 | 0.001 | 18 |
| BP | GO:0001991 | regulation of systemic arterial blood pressure by circulatory renin-angiotensin | 4/456 | 0.001 | 4 |
| BP | GO:0010544 | negative regulation of platelet activation | 4/456 | 0.001 | 4 |
| BP | GO:0034643 | establishment of mitochondrion localization, microtubule-mediated | 4/456 | 0.001 | 4 |
| BP | GO:0047497 | mitochondrion transport along microtubule | 4/456 | 0.001 | 4 |
| BP | GO:0003176 | aortic valve development | 5/456 | 0.001 | 5 |
| BP | GO:0086019 | cell-cell signaling involved in cardiac conduction | 5/456 | 0.001 | 5 |
| BP | GO:0030326 | embryonic limb morphogenesis | 10/456 | 0.001 | 10 |
| BP | GO:0035113 | embryonic appendage morphogenesis | 10/456 | 0.001 | 10 |
| BP | GO:0030308 | negative regulation of cell growth | 12/456 | 0.001 | 12 |
| BP | GO:0055002 | striated muscle cell development | 11/456 | 0.001 | 11 |
| BP | GO:0071774 | response to fibroblast growth factor | 11/456 | 0.001 | 11 |
| BP | GO:1901654 | response to ketone | 13/456 | 0.001 | 13 |
| BP | GO:0014812 | muscle cell migration | 9/456 | 0.001 | 9 |
| BP | GO:0032526 | response to retinoic acid | 9/456 | 0.001 | 9 |
| BP | GO:0001654 | eye development | 19/456 | 0.001 | 19 |
| BP | GO:0009100 | glycoprotein metabolic process | 20/456 | 0.001 | 20 |
| BP | GO:0008217 | regulation of blood pressure | 12/456 | 0.001 | 12 |
| BP | GO:0032102 | negative regulation of response to external stimulus | 19/456 | 0.001 | 19 |
| BP | GO:0010721 | negative regulation of cell development | 18/456 | 0.001 | 18 |
| BP | GO:0043270 | positive regulation of ion transport | 16/456 | 0.001 | 16 |
| BP | GO:0035637 | multicellular organismal signaling | 13/456 | 0.001 | 13 |
| BP | GO:0043393 | regulation of protein binding | 14/456 | 0.001 | 14 |
| BP | GO:0010517 | regulation of phospholipase activity | 7/456 | 0.001 | 7 |
| BP | GO:0042476 | odontogenesis | 10/456 | 0.001 | 10 |
| BP | GO:0014910 | regulation of smooth muscle cell migration | 8/456 | 0.001 | 8 |
| BP | GO:0010712 | regulation of collagen metabolic process | 6/456 | 0.001 | 6 |
| BP | GO:0030195 | negative regulation of blood coagulation | 6/456 | 0.001 | 6 |
| BP | GO:0150063 | visual system development | 19/456 | 0.001 | 19 |
| BP | GO:0045926 | negative regulation of growth | 15/456 | 0.002 | 15 |
| BP | GO:0021761 | limbic system development | 9/456 | 0.002 | 9 |
| BP | GO:1903391 | regulation of adherens junction organization | 7/456 | 0.002 | 7 |
| BP | GO:0007163 | establishment or maintenance of cell polarity | 12/456 | 0.002 | 12 |
| BP | GO:0021955 | central nervous system neuron axonogenesis | 5/456 | 0.002 | 5 |
| BP | GO:0045667 | regulation of osteoblast differentiation | 10/456 | 0.002 | 10 |
| BP | GO:0086009 | membrane repolarization | 6/456 | 0.002 | 6 |
| BP | GO:1900047 | negative regulation of hemostasis | 6/456 | 0.002 | 6 |
| BP | GO:0061515 | myeloid cell development | 7/456 | 0.002 | 7 |
| BP | GO:0086003 | cardiac muscle cell contraction | 7/456 | 0.002 | 7 |
| BP | GO:0045444 | fat cell differentiation | 14/456 | 0.002 | 14 |
| BP | GO:0010595 | positive regulation of endothelial cell migration | 10/456 | 0.002 | 10 |
| BP | GO:0051588 | regulation of neurotransmitter transport | 10/456 | 0.002 | 10 |
| BP | GO:0001574 | ganglioside biosynthetic process | 3/456 | 0.002 | 3 |
| BP | GO:0001778 | plasma membrane repair | 3/456 | 0.002 | 3 |
| BP | GO:0048484 | enteric nervous system development | 3/456 | 0.002 | 3 |
| BP | GO:0060600 | dichotomous subdivision of an epithelial terminal unit | 3/456 | 0.002 | 3 |
| BP | GO:0086070 | SA node cell to atrial cardiac muscle cell communication | 3/456 | 0.002 | 3 |
| BP | GO:0030282 | bone mineralization | 9/456 | 0.002 | 9 |
| BP | GO:0003094 | glomerular filtration | 4/456 | 0.002 | 4 |
| BP | GO:0003177 | pulmonary valve development | 4/456 | 0.002 | 4 |
| BP | GO:0008045 | motor neuron axon guidance | 4/456 | 0.002 | 4 |
| BP | GO:0048485 | sympathetic nervous system development | 4/456 | 0.002 | 4 |
| BP | GO:0055093 | response to hyperoxia | 4/456 | 0.002 | 4 |
| BP | GO:1902667 | regulation of axon guidance | 4/456 | 0.002 | 4 |
| BP | GO:0048146 | positive regulation of fibroblast proliferation | 6/456 | 0.002 | 6 |
| BP | GO:0051058 | negative regulation of small GTPase mediated signal transduction | 6/456 | 0.002 | 6 |
| BP | GO:0072132 | mesenchyme morphogenesis | 6/456 | 0.002 | 6 |
| BP | GO:0098930 | axonal transport | 6/456 | 0.002 | 6 |
| BP | GO:0003203 | endocardial cushion morphogenesis | 5/456 | 0.002 | 5 |
| BP | GO:0050679 | positive regulation of epithelial cell proliferation | 13/456 | 0.002 | 13 |
| BP | GO:0070509 | calcium ion import | 7/456 | 0.002 | 7 |
| BP | GO:0072009 | nephron epithelium development | 9/456 | 0.002 | 9 |
| BP | GO:0003231 | cardiac ventricle development | 10/456 | 0.002 | 10 |
| BP | GO:0043406 | positive regulation of MAP kinase activity | 15/456 | 0.002 | 15 |
| BP | GO:0048562 | embryonic organ morphogenesis | 16/456 | 0.002 | 16 |
| BP | GO:0002573 | myeloid leukocyte differentiation | 13/456 | 0.002 | 13 |
| BP | GO:0045665 | negative regulation of neuron differentiation | 13/456 | 0.002 | 13 |
| BP | GO:0048747 | muscle fiber development | 6/456 | 0.002 | 6 |
| BP | GO:0060193 | positive regulation of lipase activity | 7/456 | 0.002 | 7 |
| BP | GO:0010811 | positive regulation of cell-substrate adhesion | 9/456 | 0.002 | 9 |
| BP | GO:0007187 | G protein-coupled receptor signaling pathway, coupled to cyclic nucleotide second messenger | 12/456 | 0.002 | 12 |
| BP | GO:0030099 | myeloid cell differentiation | 21/456 | 0.002 | 21 |
| BP | GO:0034333 | adherens junction assembly | 8/456 | 0.002 | 8 |
| BP | GO:0050764 | regulation of phagocytosis | 8/456 | 0.002 | 8 |
| BP | GO:0033622 | integrin activation | 4/456 | 0.002 | 4 |
| BP | GO:0051654 | establishment of mitochondrion localization | 4/456 | 0.002 | 4 |
| BP | GO:0097205 | renal filtration | 4/456 | 0.002 | 4 |
| BP | GO:0045766 | positive regulation of angiogenesis | 13/456 | 0.002 | 13 |
| BP | GO:0050819 | negative regulation of coagulation | 6/456 | 0.002 | 6 |
| BP | GO:0001656 | metanephros development | 8/456 | 0.002 | 8 |
| BP | GO:0010717 | regulation of epithelial to mesenchymal transition | 8/456 | 0.002 | 8 |
| BP | GO:0030148 | sphingolipid biosynthetic process | 8/456 | 0.002 | 8 |
| BP | GO:0043473 | pigmentation | 8/456 | 0.002 | 8 |
| BP | GO:0042692 | muscle cell differentiation | 19/456 | 0.002 | 19 |
| BP | GO:0009636 | response to toxic substance | 24/456 | 0.002 | 24 |
| BP | GO:0032060 | bleb assembly | 3/456 | 0.002 | 3 |
| BP | GO:0001990 | regulation of systemic arterial blood pressure by hormone | 5/456 | 0.002 | 5 |
| BP | GO:0060306 | regulation of membrane repolarization | 5/456 | 0.002 | 5 |
| BP | GO:0007193 | adenylate cyclase-inhibiting G protein-coupled receptor signaling pathway | 7/456 | 0.002 | 7 |
| BP | GO:1990266 | neutrophil migration | 7/456 | 0.002 | 7 |
| BP | GO:0051099 | positive regulation of binding | 12/456 | 0.002 | 12 |
| BP | GO:0006909 | phagocytosis | 17/456 | 0.002 | 17 |
| BP | GO:0010594 | regulation of endothelial cell migration | 14/456 | 0.002 | 14 |
| BP | GO:0071526 | semaphorin-plexin signaling pathway | 4/456 | 0.002 | 4 |
| BP | GO:0072215 | regulation of metanephros development | 4/456 | 0.002 | 4 |
| BP | GO:0043627 | response to estrogen | 7/456 | 0.003 | 7 |
| BP | GO:0061045 | negative regulation of wound healing | 7/456 | 0.003 | 7 |
| BP | GO:1903779 | regulation of cardiac conduction | 7/456 | 0.003 | 7 |
| BP | GO:0035282 | segmentation | 8/456 | 0.003 | 8 |
| BP | GO:0072080 | nephron tubule development | 8/456 | 0.003 | 8 |
| BP | GO:0035329 | hippo signaling | 5/456 | 0.003 | 5 |
| BP | GO:0048008 | platelet-derived growth factor receptor signaling pathway | 6/456 | 0.003 | 6 |
| BP | GO:0048015 | phosphatidylinositol-mediated signaling | 11/456 | 0.003 | 11 |
| BP | GO:0070252 | actin-mediated cell contraction | 9/456 | 0.003 | 9 |
| BP | GO:0060191 | regulation of lipase activity | 8/456 | 0.003 | 8 |
| BP | GO:0086001 | cardiac muscle cell action potential | 7/456 | 0.003 | 7 |
| BP | GO:0051235 | maintenance of location | 16/456 | 0.003 | 16 |
| BP | GO:0044344 | cellular response to fibroblast growth factor stimulus | 10/456 | 0.003 | 10 |
| BP | GO:0007411 | axon guidance | 13/456 | 0.003 | 13 |
| BP | GO:0043523 | regulation of neuron apoptotic process | 13/456 | 0.003 | 13 |
| BP | GO:0086065 | cell communication involved in cardiac conduction | 6/456 | 0.003 | 6 |
| BP | GO:0042472 | inner ear morphogenesis | 8/456 | 0.003 | 8 |
| BP | GO:0003272 | endocardial cushion formation | 4/456 | 0.003 | 4 |
| BP | GO:0006688 | glycosphingolipid biosynthetic process | 4/456 | 0.003 | 4 |
| BP | GO:0072012 | glomerulus vasculature development | 4/456 | 0.003 | 4 |
| BP | GO:0051098 | regulation of binding | 19/456 | 0.003 | 19 |
| BP | GO:0060119 | inner ear receptor cell development | 5/456 | 0.003 | 5 |
| BP | GO:0007188 | adenylate cyclase-modulating G protein-coupled receptor signaling pathway | 11/456 | 0.003 | 11 |
| BP | GO:0002002 | regulation of angiotensin levels in blood | 3/456 | 0.003 | 3 |
| BP | GO:0002003 | angiotensin maturation | 3/456 | 0.003 | 3 |
| BP | GO:0032252 | secretory granule localization | 3/456 | 0.003 | 3 |
| BP | GO:0090331 | negative regulation of platelet aggregation | 3/456 | 0.003 | 3 |
| BP | GO:0021700 | developmental maturation | 15/456 | 0.003 | 15 |
| BP | GO:0050680 | negative regulation of epithelial cell proliferation | 11/456 | 0.003 | 11 |
| BP | GO:0001837 | epithelial to mesenchymal transition | 10/456 | 0.003 | 10 |
| BP | GO:0010001 | glial cell differentiation | 13/456 | 0.003 | 13 |
| BP | GO:0097485 | neuron projection guidance | 13/456 | 0.003 | 13 |
| BP | GO:0010518 | positive regulation of phospholipase activity | 6/456 | 0.003 | 6 |
| BP | GO:0042326 | negative regulation of phosphorylation | 22/456 | 0.003 | 22 |
| BP | GO:0051402 | neuron apoptotic process | 14/456 | 0.003 | 14 |
| BP | GO:0006937 | regulation of muscle contraction | 11/456 | 0.003 | 11 |
| BP | GO:0003002 | regionalization | 17/456 | 0.003 | 17 |
| BP | GO:1900120 | regulation of receptor binding | 4/456 | 0.003 | 4 |
| BP | GO:0034109 | homotypic cell-cell adhesion | 7/456 | 0.003 | 7 |
| BP | GO:0060411 | cardiac septum morphogenesis | 7/456 | 0.003 | 7 |
| BP | GO:0006790 | sulfur compound metabolic process | 18/456 | 0.003 | 18 |
| BP | GO:0046847 | filopodium assembly | 6/456 | 0.003 | 6 |
| BP | GO:0031345 | negative regulation of cell projection organization | 11/456 | 0.004 | 11 |
| BP | GO:0061041 | regulation of wound healing | 10/456 | 0.004 | 10 |
| BP | GO:0005976 | polysaccharide metabolic process | 8/456 | 0.004 | 8 |
| BP | GO:0072678 | T cell migration | 6/456 | 0.004 | 6 |
| BP | GO:0023061 | signal release | 21/456 | 0.004 | 21 |
| BP | GO:0043010 | camera-type eye development | 16/456 | 0.004 | 16 |
| BP | GO:0030258 | lipid modification | 12/456 | 0.004 | 12 |
| BP | GO:0061337 | cardiac conduction | 10/456 | 0.004 | 10 |
| BP | GO:0042659 | regulation of cell fate specification | 3/456 | 0.004 | 3 |
| BP | GO:0043116 | negative regulation of vascular permeability | 3/456 | 0.004 | 3 |
| BP | GO:0072182 | regulation of nephron tubule epithelial cell differentiation | 3/456 | 0.004 | 3 |
| BP | GO:0098903 | regulation of membrane repolarization during action potential | 3/456 | 0.004 | 3 |
| BP | GO:2001256 | regulation of store-operated calcium entry | 3/456 | 0.004 | 3 |
| BP | GO:0001708 | cell fate specification | 7/456 | 0.004 | 7 |
| BP | GO:0061333 | renal tubule morphogenesis | 7/456 | 0.004 | 7 |
| BP | GO:0007205 | protein kinase C-activating G protein-coupled receptor signaling pathway | 4/456 | 0.004 | 4 |
| BP | GO:0019934 | cGMP-mediated signaling | 4/456 | 0.004 | 4 |
| BP | GO:0032967 | positive regulation of collagen biosynthetic process | 4/456 | 0.004 | 4 |
| BP | GO:0050919 | negative chemotaxis | 4/456 | 0.004 | 4 |
| BP | GO:1902904 | negative regulation of supramolecular fiber organization | 9/456 | 0.004 | 9 |
| BP | GO:0046328 | regulation of JNK cascade | 11/456 | 0.004 | 11 |
| BP | GO:0051646 | mitochondrion localization | 5/456 | 0.004 | 5 |
| BP | GO:0099622 | cardiac muscle cell membrane repolarization | 5/456 | 0.004 | 5 |
| BP | GO:0006940 | regulation of smooth muscle contraction | 6/456 | 0.004 | 6 |
| BP | GO:0030593 | neutrophil chemotaxis | 6/456 | 0.004 | 6 |
| BP | GO:0007162 | negative regulation of cell adhesion | 15/456 | 0.004 | 15 |
| BP | GO:0050768 | negative regulation of neurogenesis | 15/456 | 0.004 | 15 |
| BP | GO:0007015 | actin filament organization | 17/456 | 0.004 | 17 |
| BP | GO:0048638 | regulation of developmental growth | 17/456 | 0.004 | 17 |
| BP | GO:0016331 | morphogenesis of embryonic epithelium | 10/456 | 0.004 | 10 |
| BP | GO:0035107 | appendage morphogenesis | 10/456 | 0.004 | 10 |
| BP | GO:0035108 | limb morphogenesis | 10/456 | 0.004 | 10 |
| BP | GO:0003151 | outflow tract morphogenesis | 7/456 | 0.004 | 7 |
| BP | GO:0006941 | striated muscle contraction | 11/456 | 0.004 | 11 |
| BP | GO:0030177 | positive regulation of Wnt signaling pathway | 11/456 | 0.004 | 11 |
| BP | GO:0097529 | myeloid leukocyte migration | 11/456 | 0.004 | 11 |
| BP | GO:0006027 | glycosaminoglycan catabolic process | 6/456 | 0.004 | 6 |
| BP | GO:0008088 | axo-dendritic transport | 6/456 | 0.004 | 6 |
| BP | GO:0033627 | cell adhesion mediated by integrin | 6/456 | 0.004 | 6 |
| BP | GO:0051893 | regulation of focal adhesion assembly | 6/456 | 0.004 | 6 |
| BP | GO:0090109 | regulation of cell-substrate junction assembly | 6/456 | 0.004 | 6 |
| BP | GO:0003044 | regulation of systemic arterial blood pressure mediated by a chemical signal | 5/456 | 0.005 | 5 |
| BP | GO:0006775 | fat-soluble vitamin metabolic process | 5/456 | 0.005 | 5 |
| BP | GO:1904646 | cellular response to amyloid-beta | 5/456 | 0.005 | 5 |
| BP | GO:0003081 | regulation of systemic arterial blood pressure by renin-angiotensin | 4/456 | 0.005 | 4 |
| BP | GO:0010714 | positive regulation of collagen metabolic process | 4/456 | 0.005 | 4 |
| BP | GO:0043410 | positive regulation of MAPK cascade | 23/456 | 0.005 | 23 |
| BP | GO:0009214 | cyclic nucleotide catabolic process | 3/456 | 0.005 | 3 |
| BP | GO:0030207 | chondroitin sulfate catabolic process | 3/456 | 0.005 | 3 |
| BP | GO:0035791 | platelet-derived growth factor receptor-beta signaling pathway | 3/456 | 0.005 | 3 |
| BP | GO:0042249 | establishment of planar polarity of embryonic epithelium | 3/456 | 0.005 | 3 |
| BP | GO:0045064 | T-helper 2 cell differentiation | 3/456 | 0.005 | 3 |
| BP | GO:0048841 | regulation of axon extension involved in axon guidance | 3/456 | 0.005 | 3 |
| BP | GO:1903034 | regulation of response to wounding | 11/456 | 0.005 | 11 |
| BP | GO:0030178 | negative regulation of Wnt signaling pathway | 12/456 | 0.005 | 12 |
| BP | GO:0030010 | establishment of cell polarity | 9/456 | 0.005 | 9 |
| BP | GO:0032965 | regulation of collagen biosynthetic process | 5/456 | 0.005 | 5 |
| BP | GO:0001933 | negative regulation of protein phosphorylation | 20/456 | 0.005 | 20 |
| BP | GO:0003073 | regulation of systemic arterial blood pressure | 7/456 | 0.005 | 7 |
| BP | GO:0019932 | second-messenger-mediated signaling | 18/456 | 0.005 | 18 |
| BP | GO:0021884 | forebrain neuron development | 4/456 | 0.005 | 4 |
| BP | GO:0036296 | response to increased oxygen levels | 4/456 | 0.005 | 4 |
| BP | GO:0042311 | vasodilation | 4/456 | 0.005 | 4 |
| BP | GO:0006026 | aminoglycan catabolic process | 6/456 | 0.005 | 6 |
| BP | GO:0021872 | forebrain generation of neurons | 6/456 | 0.005 | 6 |
| BP | GO:0045216 | cell-cell junction organization | 9/456 | 0.005 | 9 |
| BP | GO:0048736 | appendage development | 11/456 | 0.006 | 11 |
| BP | GO:0060173 | limb development | 11/456 | 0.006 | 11 |
| BP | GO:1901136 | carbohydrate derivative catabolic process | 11/456 | 0.006 | 11 |
| BP | GO:0003197 | endocardial cushion development | 5/456 | 0.006 | 5 |
| BP | GO:0010463 | mesenchymal cell proliferation | 5/456 | 0.006 | 5 |
| BP | GO:0022602 | ovulation cycle process | 5/456 | 0.006 | 5 |
| BP | GO:0046580 | negative regulation of Ras protein signal transduction | 5/456 | 0.006 | 5 |
| BP | GO:0060412 | ventricular septum morphogenesis | 5/456 | 0.006 | 5 |
| BP | GO:0060326 | cell chemotaxis | 14/456 | 0.006 | 14 |
| BP | GO:0019915 | lipid storage | 6/456 | 0.006 | 6 |
| BP | GO:0043200 | response to amino acid | 8/456 | 0.006 | 8 |
| BP | GO:0071900 | regulation of protein serine/threonine kinase activity | 22/456 | 0.006 | 22 |
| BP | GO:0031532 | actin cytoskeleton reorganization | 7/456 | 0.006 | 7 |
| BP | GO:0007204 | positive regulation of cytosolic calcium ion concentration | 14/456 | 0.006 | 14 |
| BP | GO:0034111 | negative regulation of homotypic cell-cell adhesion | 3/456 | 0.006 | 3 |
| BP | GO:0048012 | hepatocyte growth factor receptor signaling pathway | 3/456 | 0.006 | 3 |
| BP | GO:0051923 | sulfation | 3/456 | 0.006 | 3 |
| BP | GO:1901841 | regulation of high voltage-gated calcium channel activity | 3/456 | 0.006 | 3 |
| BP | GO:0021602 | cranial nerve morphogenesis | 4/456 | 0.006 | 4 |
| BP | GO:0048732 | gland development | 20/456 | 0.006 | 20 |
| BP | GO:0032330 | regulation of chondrocyte differentiation | 5/456 | 0.006 | 5 |
| BP | GO:1904888 | cranial skeletal system development | 6/456 | 0.006 | 6 |
| BP | GO:0030705 | cytoskeleton-dependent intracellular transport | 10/456 | 0.006 | 10 |
| CC | GO:0062023 | collagen-containing extracellular matrix | 54/466 | 0.000 | 54 |
| CC | GO:0031012 | extracellular matrix | 57/466 | 0.000 | 57 |
| CC | GO:0005925 | focal adhesion | 38/466 | 0.000 | 38 |
| CC | GO:0005924 | cell-substrate adherens junction | 38/466 | 0.000 | 38 |
| CC | GO:0030055 | cell-substrate junction | 38/466 | 0.000 | 38 |
| CC | GO:0005581 | collagen trimer | 17/466 | 0.000 | 17 |
| CC | GO:0005788 | endoplasmic reticulum lumen | 31/466 | 0.000 | 31 |
| CC | GO:0005912 | adherens junction | 39/466 | 0.000 | 39 |
| CC | GO:0044420 | extracellular matrix component | 12/466 | 0.000 | 12 |
| CC | GO:0098644 | complex of collagen trimers | 8/466 | 0.000 | 8 |
| CC | GO:0005604 | basement membrane | 15/466 | 0.000 | 15 |
| CC | GO:0042383 | sarcolemma | 16/466 | 0.000 | 16 |
| CC | GO:0005583 | fibrillar collagen trimer | 6/466 | 0.000 | 6 |
| CC | GO:0098643 | banded collagen fibril | 6/466 | 0.000 | 6 |
| CC | GO:0031091 | platelet alpha granule | 13/466 | 0.000 | 13 |
| CC | GO:0031674 | I band | 14/466 | 0.000 | 14 |
| CC | GO:0043292 | contractile fiber | 19/466 | 0.000 | 19 |
| CC | GO:0031252 | cell leading edge | 27/466 | 0.000 | 27 |
| CC | GO:0044449 | contractile fiber part | 18/466 | 0.000 | 18 |
| CC | GO:0030017 | sarcomere | 17/466 | 0.000 | 17 |
| CC | GO:0030016 | myofibril | 18/466 | 0.000 | 18 |
| CC | GO:0030018 | Z disc | 13/466 | 0.000 | 13 |
| CC | GO:0030426 | growth cone | 16/466 | 0.000 | 16 |
| CC | GO:0030427 | site of polarized growth | 16/466 | 0.000 | 16 |
| CC | GO:0150034 | distal axon | 20/466 | 0.000 | 20 |
| CC | GO:0033267 | axon part | 23/466 | 0.000 | 23 |
| CC | GO:0031093 | platelet alpha granule lumen | 9/466 | 0.000 | 9 |
| CC | GO:0043025 | neuronal cell body | 26/466 | 0.000 | 26 |
| CC | GO:0001726 | ruffle | 14/466 | 0.000 | 14 |
| CC | GO:0030027 | lamellipodium | 15/466 | 0.000 | 15 |
| CC | GO:0043235 | receptor complex | 19/466 | 0.000 | 19 |
| CC | GO:0015629 | actin cytoskeleton | 24/466 | 0.000 | 24 |
| CC | GO:0042641 | actomyosin | 8/466 | 0.000 | 8 |
| CC | GO:0030315 | T-tubule | 7/466 | 0.000 | 7 |
| CC | GO:0098857 | membrane microdomain | 19/466 | 0.000 | 19 |
| CC | GO:0098589 | membrane region | 19/466 | 0.000 | 19 |
| CC | GO:0032432 | actin filament bundle | 7/466 | 0.001 | 7 |
| CC | GO:0031092 | platelet alpha granule membrane | 4/466 | 0.001 | 4 |
| CC | GO:0031594 | neuromuscular junction | 7/466 | 0.001 | 7 |
| CC | GO:0045121 | membrane raft | 18/466 | 0.001 | 18 |
| CC | GO:0042734 | presynaptic membrane | 11/466 | 0.001 | 11 |
| CC | GO:0031258 | lamellipodium membrane | 4/466 | 0.002 | 4 |
| CC | GO:0045177 | apical part of cell | 19/466 | 0.002 | 19 |
| CC | GO:0048787 | presynaptic active zone membrane | 4/466 | 0.002 | 4 |
| CC | GO:0005884 | actin filament | 7/466 | 0.002 | 7 |
| CC | GO:0001725 | stress fiber | 6/466 | 0.003 | 6 |
| CC | GO:0097517 | contractile actin filament bundle | 6/466 | 0.003 | 6 |
| CC | GO:0005901 | caveola | 7/466 | 0.004 | 7 |
| CC | GO:0005874 | microtubule | 18/466 | 0.004 | 18 |
| CC | GO:0005911 | cell-cell junction | 20/466 | 0.004 | 20 |
| CC | GO:0098858 | actin-based cell projection | 12/466 | 0.005 | 12 |
| CC | GO:0097060 | synaptic membrane | 19/466 | 0.005 | 19 |
| CC | GO:0005905 | clathrin-coated pit | 6/466 | 0.006 | 6 |
| CC | GO:0008305 | integrin complex | 4/466 | 0.006 | 4 |
| MF | GO:0005201 | extracellular matrix structural constituent | 32/462 | 0.000 | 32 |
| MF | GO:0030020 | extracellular matrix structural constituent conferring tensile strength | 15/462 | 0.000 | 15 |
| MF | GO:0048407 | platelet-derived growth factor binding | 7/462 | 0.000 | 7 |
| MF | GO:0019838 | growth factor binding | 18/462 | 0.000 | 18 |
| MF | GO:0005178 | integrin binding | 16/462 | 0.000 | 16 |
| MF | GO:0005518 | collagen binding | 10/462 | 0.000 | 10 |
| MF | GO:0003779 | actin binding | 26/462 | 0.000 | 26 |
| MF | GO:0004065 | arylsulfatase activity | 5/462 | 0.000 | 5 |
| MF | GO:0015026 | coreceptor activity | 8/462 | 0.000 | 8 |
| MF | GO:0030228 | lipoprotein particle receptor activity | 5/462 | 0.000 | 5 |
| MF | GO:0005539 | glycosaminoglycan binding | 17/462 | 0.000 | 17 |
| MF | GO:0008484 | sulfuric ester hydrolase activity | 5/462 | 0.000 | 5 |
| MF | GO:0008017 | microtubule binding | 15/462 | 0.000 | 15 |
| MF | GO:0008201 | heparin binding | 14/462 | 0.000 | 14 |
| MF | GO:0002020 | protease binding | 12/462 | 0.000 | 12 |
| MF | GO:1901681 | sulfur compound binding | 17/462 | 0.000 | 17 |
| MF | GO:0001540 | amyloid-beta binding | 9/462 | 0.000 | 9 |
| MF | GO:0051015 | actin filament binding | 13/462 | 0.000 | 13 |
| MF | GO:0004222 | metalloendopeptidase activity | 10/462 | 0.000 | 10 |
| MF | GO:0015631 | tubulin binding | 18/462 | 0.000 | 18 |
| MF | GO:0005041 | low-density lipoprotein particle receptor activity | 4/462 | 0.000 | 4 |
| MF | GO:0008146 | sulfotransferase activity | 7/462 | 0.000 | 7 |
| MF | GO:0043394 | proteoglycan binding | 6/462 | 0.000 | 6 |
| MF | GO:0005161 | platelet-derived growth factor receptor binding | 4/462 | 0.000 | 4 |
| MF | GO:0050839 | cell adhesion molecule binding | 25/462 | 0.001 | 25 |
| MF | GO:0017147 | Wnt-protein binding | 5/462 | 0.001 | 5 |
| MF | GO:0071837 | HMG box domain binding | 4/462 | 0.001 | 4 |
| MF | GO:0003951 | NAD+ kinase activity | 4/462 | 0.001 | 4 |
| MF | GO:0050840 | extracellular matrix binding | 6/462 | 0.001 | 6 |
| MF | GO:0008081 | phosphoric diester hydrolase activity | 8/462 | 0.001 | 8 |
| MF | GO:0004435 | phosphatidylinositol phospholipase C activity | 4/462 | 0.002 | 4 |
| MF | GO:0016782 | transferase activity, transferring sulfur-containing groups | 7/462 | 0.002 | 7 |
| MF | GO:0008376 | acetylgalactosaminyltransferase activity | 5/462 | 0.002 | 5 |
| MF | GO:0019955 | cytokine binding | 9/462 | 0.002 | 9 |
| MF | GO:0004713 | protein tyrosine kinase activity | 10/462 | 0.002 | 10 |
| MF | GO:0017124 | SH3 domain binding | 10/462 | 0.002 | 10 |
| MF | GO:0004714 | transmembrane receptor protein tyrosine kinase activity | 6/462 | 0.002 | 6 |
| MF | GO:0042277 | peptide binding | 14/462 | 0.002 | 14 |
| MF | GO:0004112 | cyclic-nucleotide phosphodiesterase activity | 4/462 | 0.003 | 4 |
| MF | GO:0004114 | 3',5'-cyclic-nucleotide phosphodiesterase activity | 4/462 | 0.003 | 4 |
| MF | GO:0016504 | peptidase activator activity | 5/462 | 0.003 | 5 |
| MF | GO:0008237 | metallopeptidase activity | 11/462 | 0.003 | 11 |
| MF | GO:0051087 | chaperone binding | 8/462 | 0.003 | 8 |
| MF | GO:0008242 | omega peptidase activity | 3/462 | 0.004 | 3 |
| MF | GO:0030553 | cGMP binding | 3/462 | 0.004 | 3 |
| MF | GO:0001968 | fibronectin binding | 4/462 | 0.004 | 4 |
| MF | GO:0004629 | phospholipase C activity | 4/462 | 0.004 | 4 |

| **Table S5.** Results of immune signature KEGG analysis. | | |  |  |  |  |  |
| --- | --- | --- | --- | --- | --- | --- | --- |
| **ID** | **Description** | **GeneRatio** | **BgRatio** | **pvalue** | **p.adjust** | **qvalue** | **Count** |
| hsa04510 | Focal adhesion | 27/226 | 199/7925 | <0.001 | <0.001 | <0.001 | 27 |
| hsa04974 | Protein digestion and absorption | 17/226 | 95/7925 | <0.001 | <0.001 | <0.001 | 17 |
| hsa04512 | ECM-receptor interaction | 16/226 | 88/7925 | <0.001 | <0.001 | <0.001 | 16 |
| hsa04151 | PI3K-Akt signaling pathway | 31/226 | 354/7925 | <0.001 | <0.001 | <0.001 | 31 |
| hsa04611 | Platelet activation | 15/226 | 124/7925 | <0.001 | <0.001 | <0.001 | 15 |
| hsa04810 | Regulation of actin cytoskeleton | 20/226 | 213/7925 | <0.001 | <0.001 | <0.001 | 20 |
| hsa04926 | Relaxin signaling pathway | 15/226 | 129/7925 | <0.001 | <0.001 | <0.001 | 15 |
| hsa04022 | cGMP-PKG signaling pathway | 17/226 | 167/7925 | <0.001 | <0.001 | <0.001 | 17 |
| hsa00532 | Glycosaminoglycan biosynthesis - chondroitin sulfate / dermatan sulfate | 6/226 | 20/7925 | <0.001 | <0.001 | <0.001 | 6 |
| hsa04933 | AGE-RAGE signaling pathway in diabetic complications | 12/226 | 100/7925 | <0.001 | 0.001 | 0.001 | 12 |
| hsa05205 | Proteoglycans in cancer | 17/226 | 204/7925 | <0.001 | 0.002 | 0.001 | 17 |
| hsa05414 | Dilated cardiomyopathy (DCM) | 11/226 | 96/7925 | <0.001 | 0.002 | 0.001 | 11 |
| hsa04015 | Rap1 signaling pathway | 17/226 | 210/7925 | <0.001 | 0.002 | 0.002 | 17 |
| hsa05146 | Amoebiasis | 11/226 | 102/7925 | <0.001 | 0.003 | 0.002 | 11 |
| hsa04010 | MAPK signaling pathway | 20/226 | 295/7925 | <0.001 | 0.005 | 0.004 | 20 |
| hsa05165 | Human papillomavirus infection | 21/226 | 330/7925 | <0.001 | 0.007 | 0.006 | 21 |
| hsa04371 | Apelin signaling pathway | 12/226 | 137/7925 | 0.001 | 0.008 | 0.006 | 12 |
| hsa05410 | Hypertrophic cardiomyopathy (HCM) | 9/226 | 90/7925 | 0.001 | 0.014 | 0.011 | 9 |
| hsa05032 | Morphine addiction | 9/226 | 91/7925 | 0.001 | 0.015 | 0.012 | 9 |
| hsa04392 | Hippo signaling pathway - multiple species | 5/226 | 29/7925 | 0.001 | 0.015 | 0.013 | 5 |
| hsa04270 | Vascular smooth muscle contraction | 11/226 | 132/7925 | 0.001 | 0.016 | 0.013 | 11 |
| hsa04071 | Sphingolipid signaling pathway | 10/226 | 119/7925 | 0.002 | 0.023 | 0.019 | 10 |
| hsa04014 | Ras signaling pathway | 15/226 | 232/7925 | 0.003 | 0.028 | 0.023 | 15 |
| hsa04072 | Phospholipase D signaling pathway | 11/226 | 148/7925 | 0.003 | 0.035 | 0.028 | 11 |
| hsa04020 | Calcium signaling pathway | 13/226 | 193/7925 | 0.003 | 0.035 | 0.028 | 13 |
| hsa04727 | GABAergic synapse | 8/226 | 89/7925 | 0.004 | 0.036 | 0.029 | 8 |
| hsa04923 | Regulation of lipolysis in adipocytes | 6/226 | 55/7925 | 0.005 | 0.042 | 0.034 | 6 |
| hsa05163 | Human cytomegalovirus infection | 14/226 | 225/7925 | 0.005 | 0.045 | 0.036 | 14 |

| **Table S6.** Results of immune signature GSEA analysis. | |  |  |  |  |  |  |
| --- | --- | --- | --- | --- | --- | --- | --- |
| **ID** | **setSize** | **enrichmentScore** | **NES** | **pvalue** | **p.adjust** | **qvalues** | **rank** |
| EPITHELIAL_MESENCHYMAL_TRANSITION | 197 | 0.688 | 2.827 | 0.001 | 0.003 | 0.001 | 2782 |
| E2F_TARGETS | 197 | 0.527 | 2.164 | 0.001 | 0.003 | 0.001 | 5800 |
| G2M_CHECKPOINT | 193 | 0.525 | 2.155 | 0.001 | 0.003 | 0.001 | 6948 |
| INFLAMMATORY_RESPONSE | 197 | 0.498 | 2.048 | 0.001 | 0.003 | 0.001 | 3968 |
| MYC_TARGETS_V1 | 198 | 0.488 | 2.007 | 0.001 | 0.003 | 0.001 | 6175 |
| APICAL_JUNCTION | 193 | 0.473 | 1.944 | 0.001 | 0.003 | 0.001 | 4516 |
| ALLOGRAFT_REJECTION | 199 | 0.470 | 1.935 | 0.001 | 0.003 | 0.001 | 6735 |
| MITOTIC_SPINDLE | 199 | 0.469 | 1.932 | 0.001 | 0.003 | 0.001 | 4481 |
| ANGIOGENESIS | 36 | 0.586 | 1.912 | 0.001 | 0.003 | 0.001 | 1930 |
| MYOGENESIS | 198 | 0.457 | 1.879 | 0.001 | 0.003 | 0.001 | 2240 |
| IL2_STAT5_SIGNALING | 195 | 0.451 | 1.850 | 0.001 | 0.003 | 0.001 | 5627 |
| UNFOLDED_PROTEIN_RESPONSE | 109 | 0.475 | 1.849 | 0.001 | 0.003 | 0.001 | 5461 |
| HEDGEHOG_SIGNALING | 35 | 0.571 | 1.849 | 0.002 | 0.006 | 0.002 | 1865 |
| COAGULATION | 138 | 0.457 | 1.815 | 0.001 | 0.003 | 0.001 | 3412 |
| COMPLEMENT | 200 | 0.438 | 1.807 | 0.001 | 0.003 | 0.001 | 4640 |
| KRAS_SIGNALING_UP | 195 | 0.440 | 1.807 | 0.001 | 0.003 | 0.001 | 5323 |
| UV_RESPONSE_DN | 138 | 0.454 | 1.801 | 0.001 | 0.003 | 0.001 | 3224 |
| MTORC1_SIGNALING | 196 | 0.433 | 1.778 | 0.001 | 0.003 | 0.001 | 5989 |
| IL6_JAK_STAT3_SIGNALING | 87 | 0.454 | 1.723 | 0.001 | 0.003 | 0.001 | 6118 |
| TNFA_SIGNALING_VIA_NFKB | 198 | 0.408 | 1.679 | 0.001 | 0.003 | 0.001 | 5088 |
| REACTIVE_OXIGEN_SPECIES_PATHWAY | 47 | 0.468 | 1.612 | 0.004 | 0.008 | 0.003 | 4740 |
| HYPOXIA | 190 | 0.372 | 1.528 | 0.001 | 0.003 | 0.001 | 5104 |
| MYC_TARGETS_V2 | 58 | 0.424 | 1.514 | 0.009 | 0.019 | 0.007 | 5362 |
| WNT_BETA_CATENIN_SIGNALING | 42 | 0.445 | 1.501 | 0.015 | 0.027 | 0.010 | 5060 |
| APOPTOSIS | 160 | 0.366 | 1.480 | 0.003 | 0.007 | 0.003 | 2496 |
| PROTEIN_SECRETION | 95 | 0.386 | 1.478 | 0.010 | 0.020 | 0.007 | 6571 |
| SPERMATOGENESIS | 133 | 0.344 | 1.359 | 0.019 | 0.033 | 0.012 | 5145 |
| INTERFERON_GAMMA_RESPONSE | 200 | 0.324 | 1.336 | 0.013 | 0.025 | 0.009 | 7086 |
| PEROXISOME | 104 | -0.287 | -1.457 | 0.012 | 0.024 | 0.008 | 3026 |
